# Bubble-Inspired Multifunctional Magnetic Microrobots for Integrated Multidimensional Targeted Biosensing

**DOI:** 10.1021/acs.nanolett.4c03089

**Published:** 2024-10-03

**Authors:** Zichen Xu, Heng Sun, Yuanhe Chen, Hon Ho Yu, Chu-Xia Deng, Qingsong Xu

**Affiliations:** † Department of Electromechanical Engineering, Faculty of Science and Technology, University of Macau, Macau 999078, China; ‡ Cancer Center, Faculty of Health Sciences, University of Macau, Macau 999078, China; § MOE Frontiers Science Center for Precision Oncology, University of Macau, Macau 999078, China; ∥ Department of Gastroenterology, Kiang Wu Hospital, Est. Coelho Amaral 62, Macau, China

**Keywords:** Bubble microrobot, magnetic actuation, biosensing, medical imaging, targeted delivery, magnetic particles

## Abstract

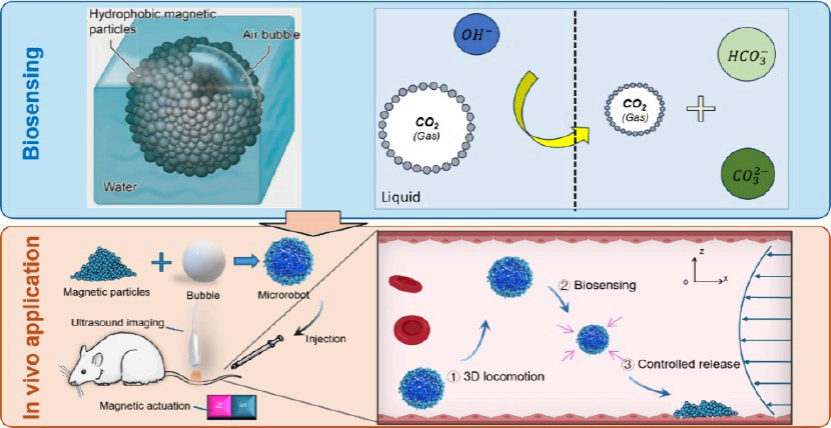

Microrobots possessing multifunctional integration are desired for therapeutics and biomedicine applications. However, existing microrobots with desired functionalities need to be fabricated through complex procedures due to their constrained volume, limited manufacturing processes, and lack of effective *in vivo* observation methods. Inspired by bubbles exhibiting various abilities, we report magnetic air bubble microrobots with simpler structures to simultaneously integrate multiple functions, including microcargo delivery, multimode locomotion, imaging, and biosensing. Contributed by buoyancy and magnetic actuation to overcome obstacles, flexible three-dimensional locomotion is implemented, guaranteeing the integrity of micro-objects adsorbed on the surface of the air bubble microrobot. Introducing air microbubbles enhances the ultrasound imaging capability of microrobots in the vascular system of mice *in vivo*, facilitating ample medical applications. Moreover, air–liquid reactions endow microrobots with rapid pH biosensing. This work provides a unique strategy to utilize relatively simple air bubbles to achieve the complex functions of microrobots for biomedical applications.

Dimension reduction of artificial systems usually results in capability sacrifices, which is attributed to the lack of accessibility of functional components, micromanufacturing processes, and sufficient volumes, especially at the microscale.^1,2^ For example, microrobots relying on material responses or mechanical performance exhibit weakened functionalities due to dimension miniaturization.^3−5^ Various microobjects with untethered actuation have been utilized as microrobots, demonstrating promising potential in reaching difficult-to-access regions within the human body for targeted therapies.^4,6−9^ They are expected to exhibit desired functions to fulfill complex microscopic assignments for practical clinical use.^10^ However, current micro/nanofabrication techniques make it difficult to achieve nm or μm-sized devices capable of onboard information processing, actuation, execution, and power supply.^11,12^ Generating desired microrobots’ functions requires complex processes where existing functional components and advances cannot be easily grafted onto them like robots on a macroscopic scale.^13−15^ Thus, it demands a new path to integrating more functions into tiny machines, ranging from basic principles to hardware implementations.^16,17^ Natural microscopic phenomena provide valuable references to exploit intrinsic intelligence to accommodate complex environments.^18,19^

In particular, natural phenomena reveal appealing physical “ingenuity”, serving as a promising source of inspiration for the design of various artificial intelligent systems.^20,21^ For example, air bubbles can scavenge biogenic organics in marine systems,^22^ enable underwater breathing for animals,^23^ and even possibly contribute to the origin of life on Earth,^24^ exhibiting intriguing abilities in matter delivery, information access, and so on, spanning from micro to macro scale. The laws of nature therein can inspire a potential path to enabling multifunctional intelligence. Currently, many microrobotic activities can be regarded as purely physical procedures.^25,26^ Elucidating and applying microscopic physical interactions of bubbles is a promising strategy for introducing the underlying natural “ingenuity” of the physical interactions into microrobot design to broaden their functionality and intelligence (Figure 1A).

**Figure 1:**
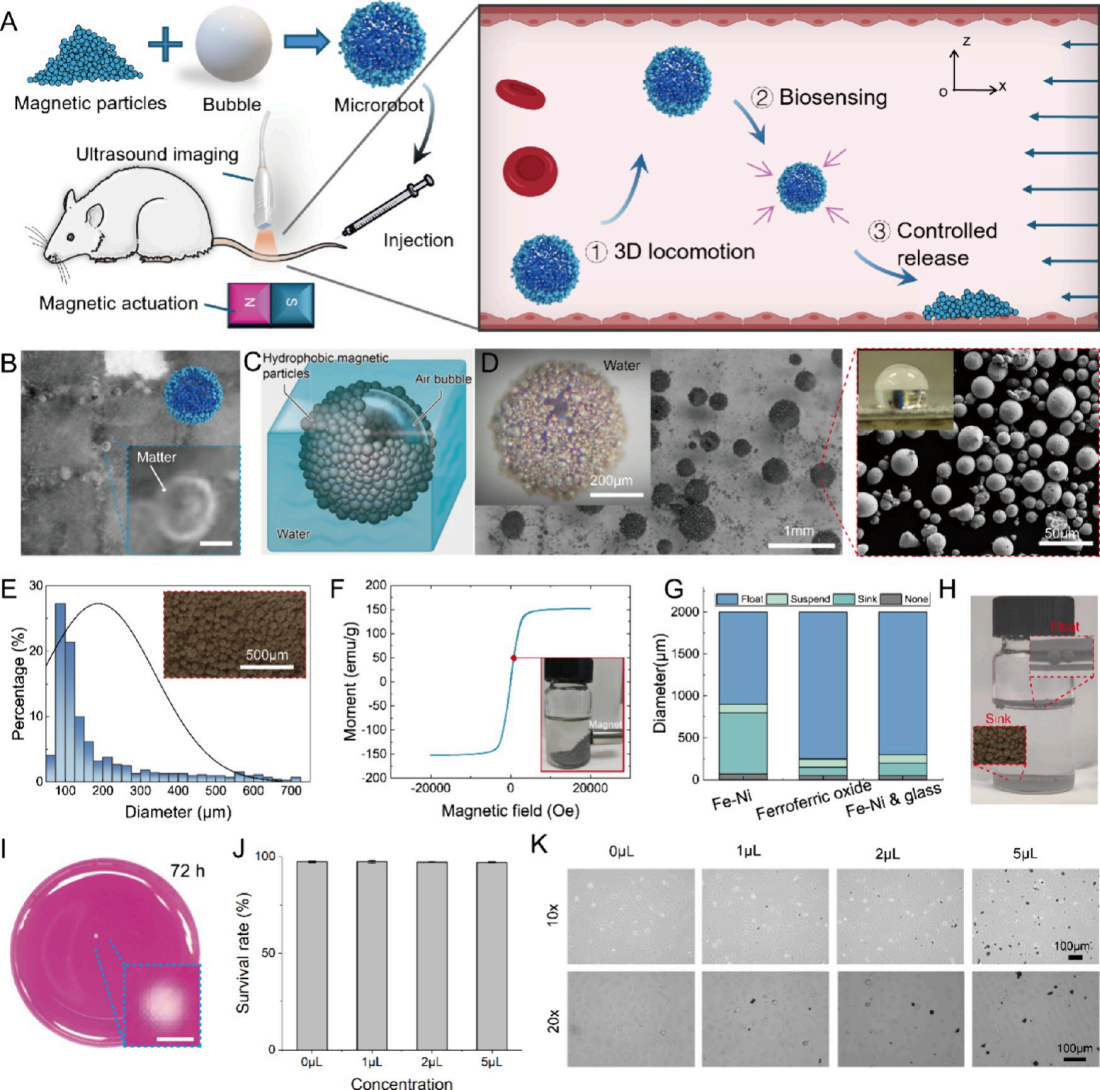
Fabrication and characterization of air bubble microrobots. (A) Schematic illustrations of the working principle and utility of the microrobot system for versatile medical applications. (B) Natural air bubbles in a pool, where matter is adhesive to the bubble’s surface (inset scale bar: 1 mm). The blue 3D model indicates bubbles with matter on the surface. (C) Schematic of a magnetic air bubble microrobot. (D) Microscope image of a magnetic air bubble microrobot. The inset indicates that numerous hydrophobic particles are adsorbed on the air bubble surface to form the microrobot, where the contact angle is around 120°. (E) Size distribution of the generated air bubble microrobots composed of air bubbles and iron–nickel alloy particles. (F) Magnetic hysteresis loop indicating the microparticle’s magnetization properties. (G) Different hydrophobic particles produce microrobots with varying relationships between sizes and densities. (H) Bigger microrobots with smaller densities float near the water–air surface. Smaller microrobots with bigger densities sink to the bottom. (I) The microrobot can remain stable in artificial blood for over 3 days (scale bar: 1 mm). (J) The survival rate of HUVEC cells (human endothelial cell line) cocultured with different magnetic particle concentrations for 48 h. (K) Experimental images of utilized HUVEC cells and magnetic microparticles.

Herein, our microrobot (100 μm or less) is constructed by μm-level magnetic hydrophobic microparticles (e.g., iron–nickel alloy) adsorbed on the bubble surface,^27,28^ also known as armored bubbles (Figure 1B–D). By leveraging relatively simple bubble structures, we demonstrated that such a unique microrobot design strategy enables stunning performance in microcargo delivery, medical imaging, and biosensing. First, hydrophobic objects are well captured for microcargo delivery by water–air interface interactions, guaranteeing cargo integrity during navigation in a flowing medium. When applying a 100-mT level of magnetic field, the microparticles are separated from the air bubble for microcargo release. This method is reliable for delivering and releasing microparticles in fast-flow environments. The air-bubble microrobot also promotes the efficiency of ultrasound observation, facilitating *in vivo* applications. By selecting proper hydrophobic particles and gases, with the help of several gas–liquid reactions, the microrobots’ shapes or structures are correlated with the fluidic environmental properties, such as the pH value, which enables biosensing under ultrasound imaging. We found that bubbles are essential components of microrobots to endow their intelligence and multifunctional integration.

The microrobot is essentially a kind of armored bubble^29−31^ (100 μm level) with magnetic hydrophobic particles (iron–nickel alloy, Figures S1–S3) adsorbed on the air bubble surface (Figure 1C,D, Section S1). By controlling the bubble generation, commonly through shaking, microrobots can be fabricated with various sizes (Figure 1E, Section S2). Unlike traditional functional bubbles,^31^ we focus more on robotic properties to leverage such a design, promoting its controllability, precision, and related functions. Introducing magnetic elements enables desired magnetic actuation, which is the basis of the robotic functionalities (Figure 1F). The inset of Figure 1F well demonstrates the magnetic responses. The microparticles’ sizes also affect the microrobots’ generation, where smaller particles produce small microrobots down to 50 μm-level (Figure 1G, Figure S4). Utilizing nanoscale particles, the microrobot’s size can be further minimized for broader applications. The mass of microrobots is mainly governed by the adsorbed layer of particles on the surface, where the microparticles can be regarded as a thin film whose thickness is constant, i.e., the diameter of particles. Thus, microrobots with different sizes exhibit different overall densities, leading to microrobots’ floating and sinking locomotion (Figure 1G,H, Section S3). Relatively large (diameter >0.8 mm) microrobots composed of iron–nickel alloy float near the water–air surface, and relatively small (diameter <0.7 mm) microrobots sink to the bottom (Figure 1H, Section S3). Unlike normal fragile bubbles that easily evanesce, the proposed armored bubble can stay stable in artificial blood for over 72 h (Figure 1I). The microrobot can remain stable for over 30 min in acidic environments (e.g., pH = 1.5) and for over 3 days in alkaline environments (e.g., pH = 12), demonstrating its attractive adaptivity in various human biofluids (Figure S5). In addition, the hydrophobic particles adsorbed on the air bubble surface enhance the stiffness of the microrobot, enabling contact manipulation of small objects (Figure S6, Movie S1). It can stay stable and maintain its structure in water for over 10 days in room conditions. Even without water, after air drying at a temperature of approximately 25 °C, the microrobot can preserve the basic spherical shell structure. By adding water, the dry structure recovers to its initial state. The microrobot can move, break, and release particles (Figure S7). Additionally, we cocultured the human endothelial cells HUVEC and murine endothelial cells SCVE4–10 with different concentrations of magnetic particles for 48 h. No significant difference was found among the cells of different experimental settings. Hence, the biosafety of microrobots is well demonstrated (Figure 1J,K, Figure S8).

To reveal the performance of magnetic actuation methods in different application scenarios, we conducted simulation and experimental studies. Usually, wireless magnetic actuation is enabled by magnetic torques via rotating magnetic fields or magnetic forces through gradient magnetic fields (Figure 2A,B). The actuation method should be chosen based on the surroundings.^32^ Smaller microrobots (diameter <0.7 mm) with larger densities sinking to the bottom have great contacts on walls. Thus, rotation (enabled by magnetic torques) and translation (induced by magnetic forces) are the desired movements of microrobots (Figure 2C,E). However, due to the lack of contact with the terrain surface for relatively light microrobots floating upward, we prefer to apply proper magnetic forces rather than magnetic torques for driving (Figure 2D,F, Section S4, Figures S9 and S10). We found that a weak magnetic field (10 mT level) can actuate the microrobots (Figure 2G,H). A cylindrical NdFeB permanent magnet (diameter: 30 mm, height: 30 mm) provides the desired gradient magnetic fields (Figure S11). When a stronger magnetic field (>30 mT) is applied, the microrobots’ structure and shape will be changed (Figure 2I, Movie S2). In fact, high energies are required to remove particles and separate them (Section S5). Accordingly, we can apply powerful magnetic fields to actuate microrobots to navigate upstream efficiently without destroying the microrobots’ structure (Figure 2J). For the microrobots that come into contact with the terrain, electromagnetic coils are used to provide a rotating magnetic field, which can offer more precise navigation control (Figure 2K, Figure S12). Microrobot’s velocity responses are governed by their density and the frequency of external magnetic fields (Figure 2L). Magnetic fields with higher frequencies lead to faster movement of microrobots. However, an excessive frequency larger than the step-out frequency will stop the microrobot (Figure 2M). All those results confirm that the microrobots’ movement can be flexibly controlled by wireless magnetic fields.

**Figure 2:**
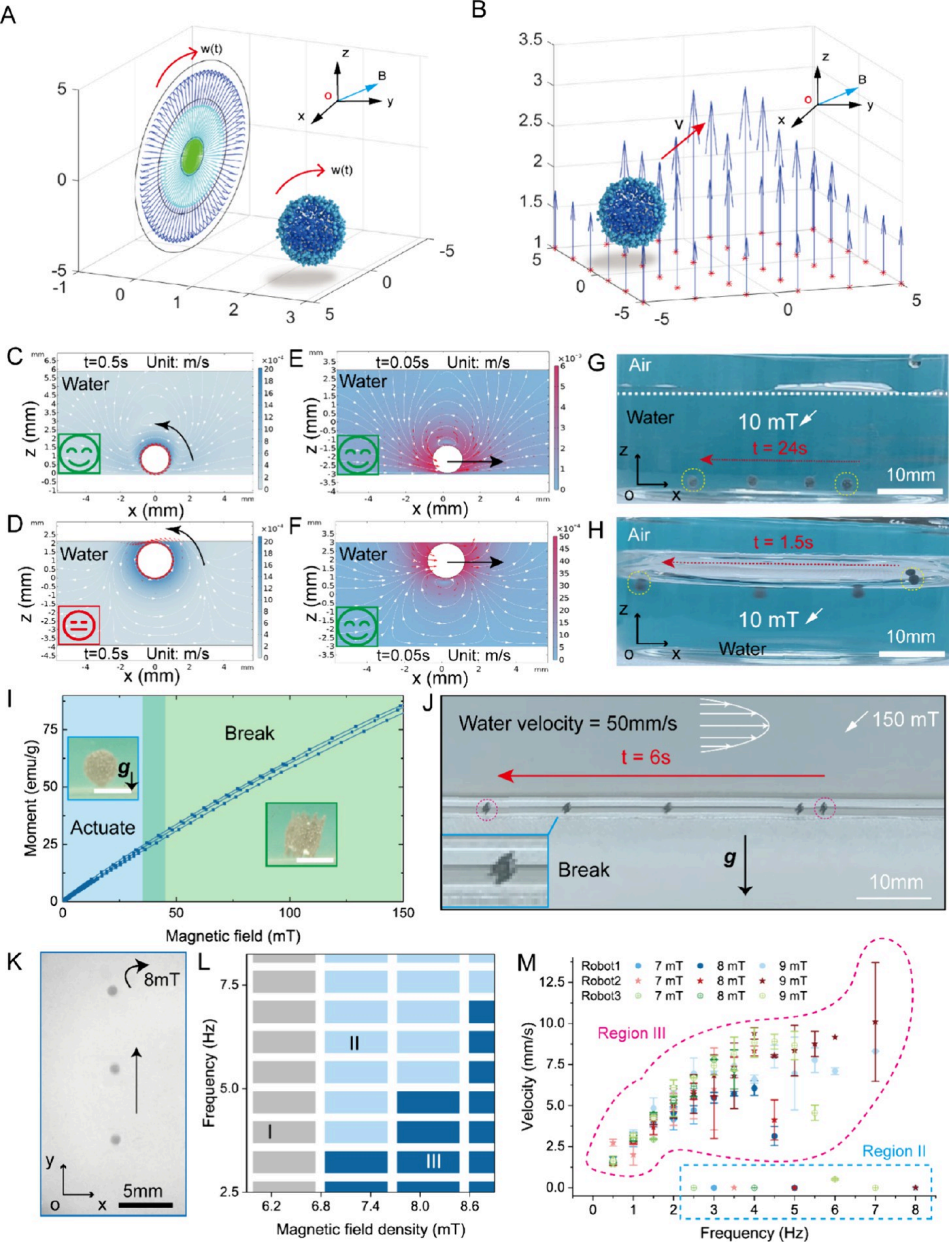
Magnetic actuation of magnetic air bubble microrobots. (A) Schematic of the microrobot’s controlled movement under rotating magnetic fields. The blue, light-blue, and green arrows indicate the magnetic field with different strengths. (B) Schematic of the microrobot’s controlled movement under gradient magnetic fields. (C) Simulation result of controlled rotation movement near the glass surface underwater. (D) Simulation result of controlled rotation movement near the water–air interface. (E) Simulation result of controlled parallel movement near the glass surface underwater. (F) Simulation result of controlled parallel movement near the water–air interface. (G) Experimental results of controlled parallel movement of the microrobot near the glass surface underwater (scale bar: 10 mm). (H) Experimental results of controlled parallel movement of the microrobot near the water–air interface (scale bar: 10 mm). (I) Utilized magnetic particles’ responses to external magnetic fields, contributing to breaking the microrobot’s structures (scale bar: 1 mm). (J) Magnetic actuation enables the microrobot to travel upstream against the flowing medium at 50 mm/s in a glass tube (inner diameter: 1.3 mm). (K) Experimental results of controlled rotation movement near the glass surface underwater (scale bar: 5 mm). (L) Phase diagram of weak rotating magnetic fields to actuate the microrobot. In Region I, the microrobot cannot be actuated. In Region II, the microrobot robot cannot move smoothly due to the rotating magnetic fields with high frequency. In Region III, the microrobot can be well actuated under the water. (M) Velocity responses to external rotation magnetic fields of three microrobots with different sizes.

To guarantee that the microrobot can reach designated positions, flexible 3D navigation is vital. The proposed microrobots can reach desired places and prevent sticking on the human tissue surfaces caused by mucus and cilia (Figure 3A). The effectiveness of the movement in gravity direction is verified in artificial blood by controlled movement (Figure 3B). For diverse medical use, microrobots are expected to carry and deliver various cargoes. To load cargoes, we mainly utilized interface interactions on the bubble surfaces, which help to adsorb a layer of hydrophobic objects. Applying different magnetic fields, magnetic particles adsorbed on the bubble surface are organized following the direction of magnetic fields. Thus, the internal air bubble is exposed to external environments to adsorb desired hydrophobic cargoes (Figure 3C, Section S6). Strong magnetic fields and intense changes allow the separation of the air bubble and magnetic particles, namely, cargo release (Figure 3D). We applied a strong magnetic field (around 100 mT) to expose the air bubble, which succeeded in adsorbing glass bead stably (Figure 3E). After that, the microrobot can flexibly carry the cargo to achieve flexible 3D navigation (Figure 3F, Movie S3). Concerning specific application scenarios, such as in the digestive system, the mucus and cilia on the tissue surface severely hinder microrobot navigation and cargo delivery. To overcome these issues, we utilize a relatively light microrobot that can float near the water–air interface. The navigation avoids contact with the tissue surface and enhances delivery efficiency. Once arriving at the top of the target position, we utilize a strong magnetic field (about 150 mT) to release bubbles and unload the particles on the target surface (Figure 3G, Movie S4). The controlled release procedure is revealed in Figure 3H. When the applied magnetic field is 150–200 mT, the microrobot structure starts to break. Finally, the particles are separated from the air bubble, completing the designated particle release process (Movie S5). In fact, the bubbles serve as a carrier with 3D navigation to achieve target delivery and on-demand release of microparticles and cargo. Surprisingly, the individual loss is almost zero. Except for the air bubble, no extra material is required. It is a brand-new strategy that exploits the physical intelligence of bubbles.

**Figure 3:**
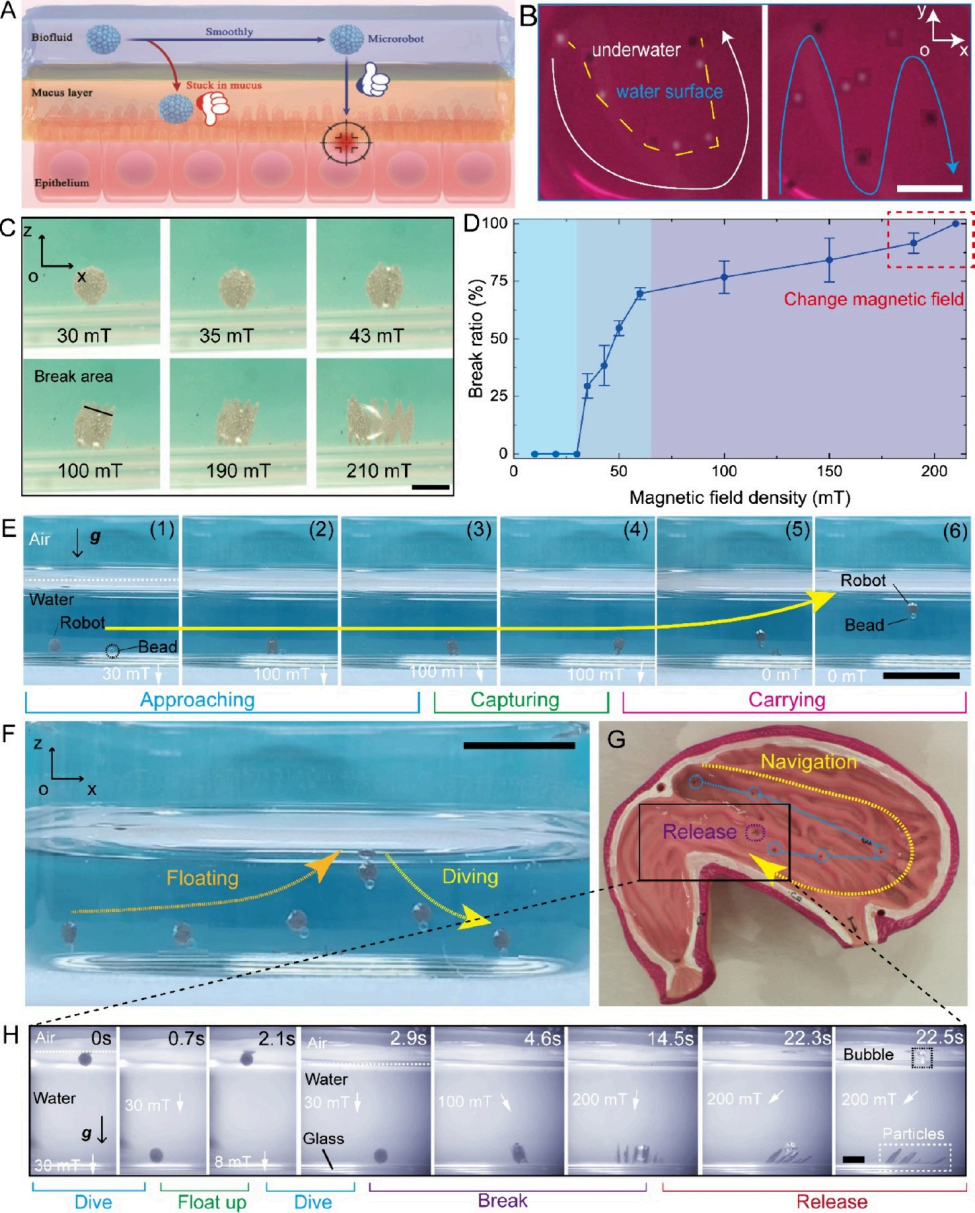
Controlled cargo delivery and on-demand release in extreme 3D environments. (A) Schematic of microrobot navigation in biofluidic environments with mucus and cilia. (B) Controlled 3D navigation of the microrobot along “UM” characters in artificial blood. The microrobot’s diameter is approximately 0.8 mm (scale bar: 5 mm). (C) Photos of a microrobot under different magnetic fields (scale bar: 1 mm). Changing the directions of magnetic fields allows the separation of the magnetic microparticles and the air bubble. (D) Shape and structure changes of the microrobot under different magnetic field densities. (E) A microrobot captures a glass bead and carries it to float upward (scale bar: 10 mm). (F) Microrobot is carrying micro-objects to achieve flexible 3D navigation (scale bar: 10 mm). (G) Microrobot navigates smoothly in the stomach model and releases particles in designated locations under complex terrain conditions. (H) Controlled movement of the microrobot in the direction of gravity and release of the surface layer of magnetic hydrophobic microparticles (scale bar: 2 mm).

Biosensing is crucial for diagnosis application. It is hard to conduct in situ sensing in deep corners of the human body. Our microrobots can reach those corners and conduct efficient biosensing in constrained space. The volume of a gas is easier to change than a liquid or solid. On this basis, the deformable bubbles can serve as microsensors to reflect the properties of fluidic environments. Herein, we utilize the liquid–gas reaction between CO_2_ gas and NaOH solution to design microrobot-based biosensors (Figure 4A), facilitating the obtaining of detailed pH value distribution within the human body, such as digestive systems. The contact between CO_2_ gas bubble and alkaline fluidic surroundings contributes to microrobots’ shape changes. The adsorbed magnetic particles reduce the contact. Thus, fully armored microrobots demonstrate slower shape change velocity in the same alkaline environment compared to partly armored microrobots (Figure 4B). Surely, the sizes of microrobots also need to be considered in actual pH sensing. The above results have shown that the contact between internal air bubbles and external fluidic environments can be well controlled by exerting magnetic fields. Hence, we can deploy the microrobot in the desired place and then expose the air bubble for biosensing, which is a potential sensing strategy to avoid unnecessary reactions during navigation. Shape change speed can also reflect the strength of alkaline environments, which helps to derive pH values. Mild alkaline environments lead to slower shape change speed (Figure 4C, Movie S6). By observing the shapes of microrobots, we can infer the pH values of fluidic environments. In addition, the applicability of biosensing can be enhanced by selecting different gases.^29−31^ For further applications *in vivo*, practical observation is essential. Considering air bubbles’ impressive performances in ultrasound imaging, the proposed air bubble microrobots can perform well in enhancing ultrasound imaging resolution for medical imaging applications (Figure 4D). In a soft silicone channel full of water, 400 μm microrobots can be observed in real-time (Figure 4E) by ultrasound equipment in B-mode. Thanks to the bubble-based structure, routine ultrasound imaging is feasible to detect microrobot position in the rigid-body plastic channel full of water, although rigid-body structures severely reduce the imaging effects (Figure 4F, Movie S7). Based on these results, we successfully utilized ultrasound imaging to capture microrobot shape changes in alkaline environments, which is a reliable pilot study for further *in vivo* sensing applications (Figure 4G). After adding numerous microrobots, the light shades and red areas are increased, which is caused by the introduction of air bubble-based structures, i.e., the proposed microrobots, as illustrated in Figure 4G (0 and 17.7 s). Comparing Figure 4G (17.7 s) and Figure 4G (236.3 s), the alkaline environment-induced microrobot shape changes are well presented. These findings confirm bubbles’ promising advantages of functional promotion, especially in imaging and sensing.

**Figure 4:**
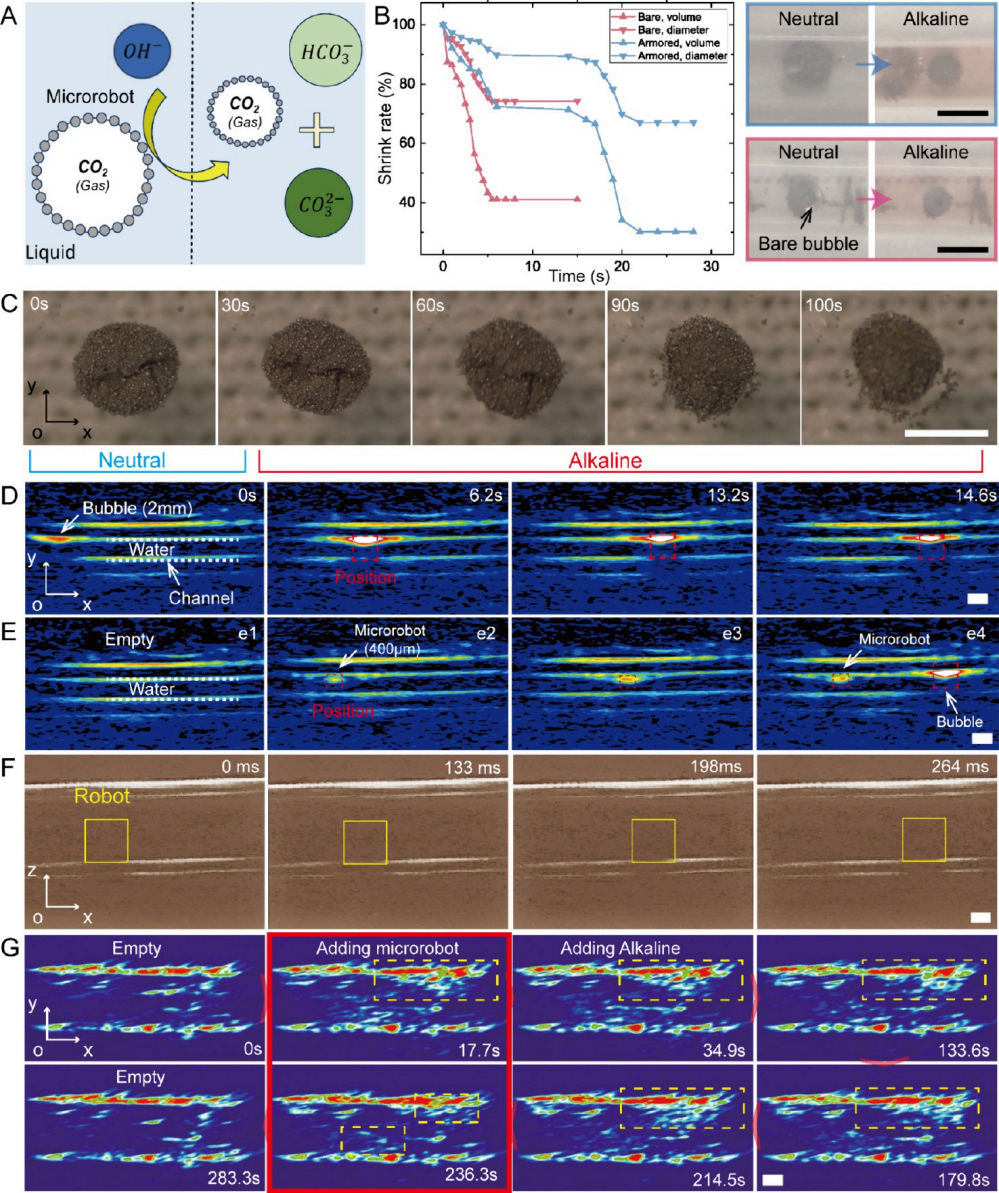
Biosensing and efficient ultrasound imaging of microrobots ex vivo. (A) Schematic of gas–liquid reaction for microrobots’ biosensing. (B) Shrink rates of microrobots with different structures change with time. The insets indicate experimental images of the fully armored microrobot shape changes and the partly armored microrobot. (C) Biosensing test results. (D) Ultrasound imaging reveals the position of an air bubble with a diameter of 2 mm in a soft silicone channel. (E) Ultrasound imaging reveals the position of the magnetic air bubble microrobot with a diameter of 400 μm in a soft silicone channel. (F) In a rigid-body plastic channel, the proposed microrobot can be captured by ultrasound imaging. (G) Utilizing ultrasound imaging to capture the biosensing procedures. The pH value change-induced microrobot shape change can be detected by ultrasound imaging. Numerous microrobots were used in the experiment. The increment of red regions and light shades indicated the addition of microrobots. Alkaline environments make microrobots small, which are clearly observed in ultrasound images. Related changes are mainly presented in the yellow dashed box. All scale bars are 1 mm.

To further reveal the advantages of the microrobots, experiments were conducted *in vivo* in a mouse’s tail vein (Figure 5A,B). The tail vein’s diameter is approximately 300 μm, where we injected the microrobots (diameter: 100 μm). First, the tiny microrobots are observed in a glass microtube through ultrasound testing equipment. It helps to obtain and reveal the features of microrobots during ultrasound imaging. Without preprocessing the ultrasound testing equipment and microrobots, 100 μm-level objects can be clearly observed in real-time (Figure 5C). Although shadow artifacts occur when sound waves are blocked by dense objects (such as the glass tube wall, resulting in signal loss and shadows on the image), the air bubble-based structure provides outstanding imaging effects under the ultrasound testing equipment. It leads to clear shades due to the presence of an air bubble that prevents ultrasound propagation. Figure 5C(c1,c2) further verifies the advantages of air bubble-based structures when processing initial ultrasound images. The red region can show the approximate position of the utilized glass tube. The shades indicate the position of microrobots. Based on these phenomena, we compared the ultrasound images of a live mouse’s tail and a dead mouse’s tail with a microrobot injected (Figure 5D,E). The microrobot can be intuitively presented, including its position information. Then, a microrobot was injected into the live mouse’s tail, where the microrobot could be clearly observed and successfully stopped in designated places in flowing blood (Figure 5F). The enhancement of ultrasound imaging quality brought by the proposed microrobot is depicted in Figure 5G. The experimental test was conducted with four additional mice, and similar data were obtained. These results justify the function of medical imaging for the proposed microrobot. For the first time, we utilized ultrasound testing equipment to capture a tiny object (down to 100 μm) in the flowing blood of a live animal in real-time (Movie S8).^6,9^ Existing microrobot swarms are almost impossible due to the loss of particles and complex fluidic environment *in vivo*. In addition, air bubble structures can be promisingly utilized to achieve active and precise air embolism and generate desired animal models.

**Figure 5:**
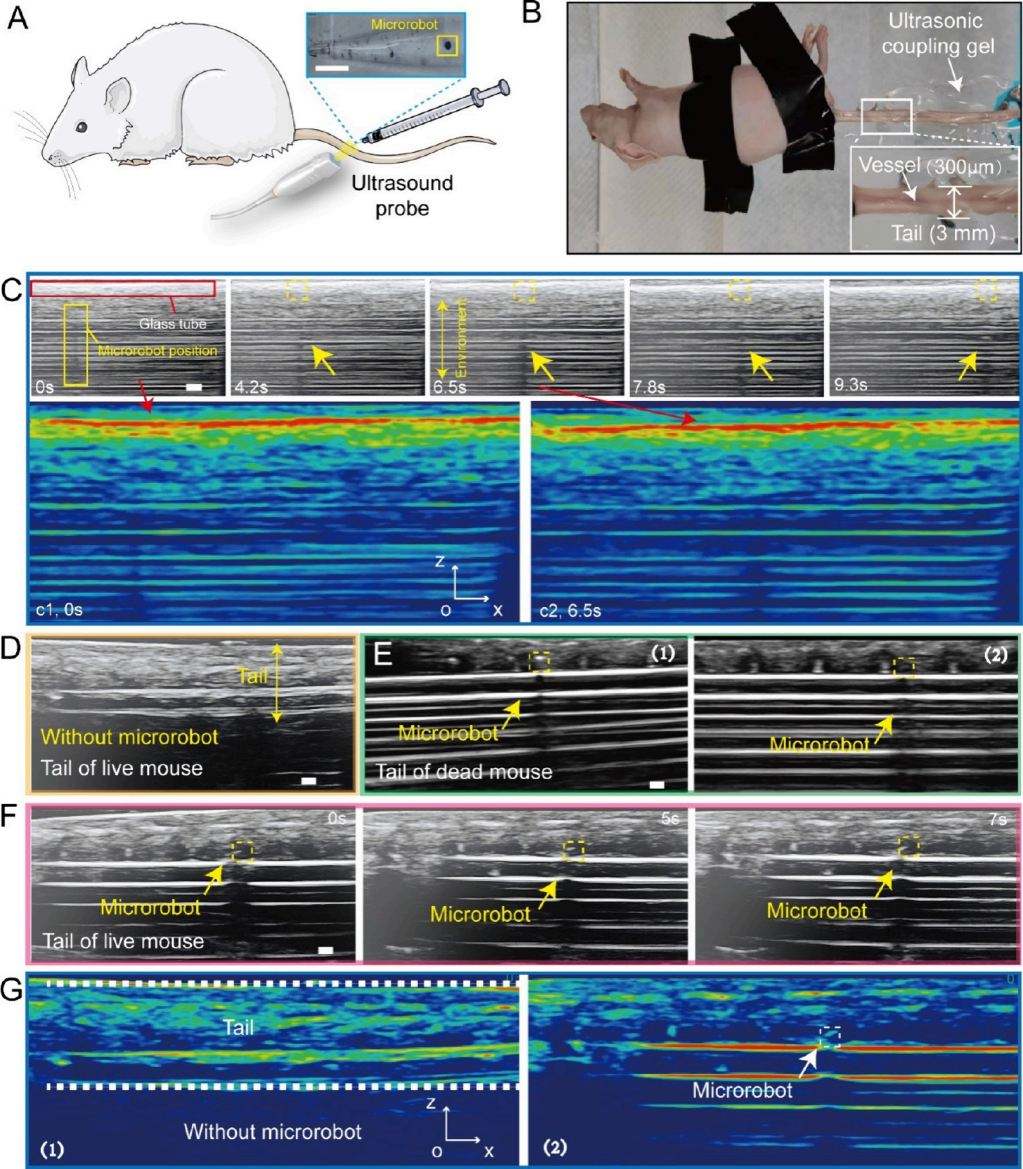
Microrobot real-time medical imaging *in vivo*. (A) Schematic of microrobot injection and ultrasound imaging for a mouse. (B) Experimental image of the utilized mouse. (C) A 100 μm air bubble microrobot’s ultrasound imaging in a micro glass tube with an inner diameter of 800 μm. (D) Ultrasound imaging of the tail of a live mouse. (E) Air bubble microrobot was clearly observed by ultrasound imaging in the dead mouse’s tail vein. Images (1) and (2) represent different positions in the dead mouse tail. (F) Air bubble microrobot was clearly observed by ultrasound imaging in the live mouse’s tail vein, which can also be stopped by an external magnetic field in the flowing blood. (G) Processed ultrasound images reveal the differences caused by microrobot’s bubble structure in the mouse tail vein. Image (1) is the ultrasound image of the utilized mouse tail without microrobots. Image (2) indicates that the microrobot was successfully injected into the tail, contributing to the intuitive shade during ultrasound imaging. All scale bars are 500 μm.

The size reduction and multifunctional intelligence of microrobots are primary pursuits of current microrobotic research.^33−35^ The ultimate goal is to integrate multifunctionalities in tiny and limited-volume microrobots as much as possible, spurring the development of related fields ranging from materials to designs.^36−39^ Our idea is to simplify the microrobot fabrication and integrate sufficient functionalities simultaneously. Although such a thought seems like a paradox of self-contradiction, the attractive performance of bubble-based design is achieved. Exploiting the underlying physical intelligence of bubbles, the simple design enables microrobots’ locomotion, delivery, medical imaging, and biosensing capabilities. The manufacturing process can be simplified into one step quickly (at a minute level). Such design enables desired functionalities in tiny enough sizes (100 μm or less) at a low cost without severe reliance on expensive micromanufacturing equipment.^38,39^

The magnetic air bubble microrobot is essentially a “minibus” that carries magnetic microparticle swarms.^13,40^ Strong magnetic fields release the microparticles, providing a remote-controlled release mechanism (Figure S13). With the help of bubbles, the movement of microrobot swarms is more flexible in fluidic environments, which is successfully extended to 3D scenarios. In contrast, for traditional magnetic microrobot swarms, flexible 3D navigation is difficult,^5,25^ exhibiting severe reliance on terrain surfaces. It is a promising method for simultaneously delivering multiple microcargoes (such as drugs and contrast agents) in biofluid environments by preventing individual loss and promoting delivery efficiency.

Leaving aside magnetic particles, the physical properties of the bubbles themselves lead to many functional enrichments.^41^ Air bubbles have a significant effect on ultrasound propagation. The bubbles and their movement can be easily detected using ultrasound imaging equipment, even for microbubbles down to 100 μm. Introducing the bubbles dramatically improves the precision and efficiency of ultrasound imaging for tiny objects. Using ultrasound imaging equipment, we first achieved real-time imaging of 100 μm-sized microrobots in the flowing blood of five live mice. Excitingly, these novel microrobots are promising biosensors that can reflect the fluidic environment within the human body because gas bubbles are sensitive to external environments. For example, gas–liquid reactions change the shapes of microrobots, which are easily observed by ultrasound testing equipment. This finding suggests that the sensing procedure is helpful for clinical use in practice. In addition, different gas bubbles can be adopted to monitor other environmental properties. For instance, the pressure-sensitive gas (NO_2_) is promising to reflect the pressure status. Furthermore, the air bubble can be integrated into more microrobot designs.

Generally, air bubbles are dangerous in vascular systems and might cause deadly gas embolisms. Due to the small sizes of microrobots, which are often less than 1 mm, the bubble volume is acceptable for medical use. Indeed, it is possible to select a proper gas (such as CO_2_) that humans can naturally adsorb to prevent potential dangers. The utilized microparticles should also fulfill the requirement of biocompatibility and safety. The better choice of magnetic microparticles and gas bubbles will further promote the performance of the proposed concept design. As a well-controlled bubble, the microrobot design paves an exciting path to achieve embolisms in the vessel (Figure S14) actively.

In summary, this work offers a novel paradigm for microrobot development that exploits physical “ingenuity” to enrich microrobotic functionalities. The design ideas are the main contribution to the microrobotics community. Physical interactions or properties at a small scale are potential components of the microrobot design for functional enrichments and intelligence enhancements. In addition to material properties, biological factors, and microfabrication techniques, such a new microrobot concept design advances microrobot research. In the future, we will further exploit the physical intelligence of microrobots to enable precise clinical applications and enhance existing microrobot performance attributes ranging from theory to application.

## Data Availability

All data are available in the main text and/or the Supporting Information.

## Supporting Information Available

Supplementary Sections S1–S6 (experimental details and physical analysis); Supplementary Figures S1–S14 (material characterization, simulation results, and experimental setup) (PDF)
Microbot manipulation (MP4)
Microbot drive and crush (MP4)
Microbot capture and carry (MP4)
Microbot navigation (MP4)
Microbot dive, float up, and break (MP4)
Microbot biosensing (MP4)
Microbot ultrasound imaging (MP4)
Microbot ultrasound imaging (glass tube; dead mouse tail) (MP4)
