# Supplementary material for: Bubble-Inspired Multifunctional Magnetic Microrobots for Integrated Multidimensional Targeted Biosensing
Source: Nano Lett. 2024 Oct 3;24(44):13945–54. doi: 10.1021/acs.nanolett.4c03089 (PMC11544691; doi:10.1021/acs.nanolett.4c03089)
Supplement: Supplementary file 1 — nl4c03089_si_001.pdf [file nl4c03089_si_001.pdf]

Supporting Information

Bubble-Inspired Multifunctional Magnetic Microrobots for Integrated Multidimensional Targeted Biosensing

Zichen Xu[1#], Heng Sun[2,3#], Yuanhe Chen[1], Hon Ho Yu[4], Chu-Xia Deng[2, 3]*, Qingsong Xu[1]*

[1]Department of Electromechanical Engineering, Faculty of Science and Technology, University of Macau, Macau, China

[2] Cancer Center, Faculty of Health Sciences, University of Macau, Macau, China

[3] MOE Frontiers Science Center for Precision Oncology, University of Macau, Macau, China

[4] Department of Gastroenterology, Kiang Wu Hospital, Est. Coelho Amaral 62, Macau, China

# These two authors contributed equally.

* Co-corresponding authors. Email: cxdeng@um.edu.mo (C. Deng); qsxu@um.edu.mo (Q. Xu)

This PDF file includes:

Section S1. Experimental section.

Section S2. Analysis of the generation of microrobots.

Section S3. Analysis of microrobot's physical parameters.

Section S4. Analysis of the microrobot actuation in fluidic environments.

Section S5. Analysis of microrobot's stability under magnetic actuation.

Section S6. Interface interactions between the magnetic air bubble microrobots and various objects.

Supplementary Figures:

Fig. S1. TEM images of the adopted magnetic microparticles.

Fig. S2. Magnetic hysteresis loop indicating the microparticle's magnetization properties.

Fig. S3. Radius distribution of the adopted microparticles in Fig. S1.

Fig. S4. Utilizing microparticles with different sizes to generate air bubble microrobots.

Fig. S5. Microrobots stay stable in various environments.

Fig. S6. Microrobot manipulates a glass bead (diameter: 0.8 mm) and places a microbubble on the bead surface.

Fig. S7. Microrobots (diameter: around 1 mm) maintain their physical structures in a dry environment and perform with the same properties as in a fluidic environment after adding water.

Fig. S8. Experimental results of biosafety testing.

Fig. S9. COMSOL simulation results of the microrobot's parallel movement under different fluidic surroundings.

Fig. S10. COMSOL simulation results of the microrobot's self-rotation under different fluidic surroundings.

Fig. S11. Detailed information of the utilized NdFeB permanent magnet.

Fig. S12. Electromagnetic coils and control computer to achieve precise movement control for micro**robots.**

Fig. S13. Wireless magnetically controlled release of magnetic particles.

Fig. S14. Controlled bubble release to form potential embolism.

Fig. S15. Images of the generated magnetic air bubble microrobots.

Fig. S16. Experimental setup for ultrasound-based guidance of the microrobot.

Fig. S17. Due to swift bubble generation by using sparkling water, microrobots are produced more efficiently than utilizing pure water.

Fig. S18. CO2 bubbles' sizes are sensitive to alkaline environments.

Other Supplementary Materials for this manuscript include the following:

Supplementary Movies

Movie S1. Microrobot manipulates tiny glass beads (diameter: 1 mm).

Movie S2. A 10-mT magnetic field can actuate the microrobots in a designated direction, and a 100-mT magnetic field is adopted to break the microrobot's structure.

Movie S3. Microrobot captures a small glass bead and carries it to realize 3D navigation.

Movie S4. Microrobot navigates smoothly in the stomach model and releases microparticles in a designated site under complex terrain conditions.

Movie S5. Microrobots with densities lower than water can navigate in the direction of gravity, providing a promising method to deliver microparticles.

Movie S6. Biosensing demonstration of a microrobot composed of CO_2 gas, which experiences shape change in alkaline environments.

Movie S7. Ultrasound imaging-guided locomotion of the microrobot.

Movie S8. Ultrasound imaging of the microrobot in a micro glass tube, a dead mouse's tail, and a live mouse's tail.

Supplementary Text

Section S1. Experimental section

Microrobot Fabrication: The magnetic microparticles were composed of 300-mesh iron-nickel alloy, and they were purchased from the Shanghai Alloy Powder Science Research Center (Figs. S1–S3). The outstanding magnetization of these microparticles was critical for achieving robotic tasks (Fig. S2). Without processing, we added 1~2 g microparticles in a small vial (3 mL or 5 mL). Then, we added approximately 2 mL of pure water to the vial and shook it by hand for 30 seconds. After placing the suspension for 1~2 minutes, we observed several magnetic air bubble microrobots. All operations were conducted at a room temperature of 25 °C. In addition, we injected the desired gas at the bottom of the suspension under the accumulation particles to produce microrobots with different gases. Because we did not perform hydrophobic treatment for the particles in advance, very few microrobots were produced (at most 5%). Hence, microrobots were easily obtained individually rather than as accumulated foams (Fig. S15). To make smaller microrobots, we can utilize smaller hydrophobic nanoparticles, where ultrasonic vibration also leads to smaller bubbles to adsorb the nanoparticles (Fig. 2**F**).

Stability of Microrobots Under Magnetic Actuation: Once microparticles are adsorbed on the bubbles' surface, high energies are required to remove particles and separate them. The microrobots can stay stable under low-energy conditions. For simplification, we only focus on the bubbles and single particles. The attachment forces (F_A) contain buoyancy (F_b), capillary (F_c), and hydrostatic pressure (F_h) induced forces. The detachment forces (F_D) contain gravity force (F_g) and capillary pressure-induced force (F_{cp}) and viscous drag force (F_d). When F_A and F_D reach the balance state, the microrobot will stay stable *(27,28,41)*. Under magnetic actuation, the introduction of magnetic forces will affect the balance between F_A and F_D. By applying an external magnetic field, those particles are magnetized, which can be regarded as tiny magnets. When the magnetic forces between magnetic particles are sufficient to break the balance, the structures of microrobots will be changed. Then, the interaction force between two particles in a magnetic field can be defined below.

$$\boldsymbol{F_{mr}} = \frac{3\mu_0 m^2}{4\pi r^4}(3\cos^2\alpha - 1) \qquad (6)$$

$$F_{mo} = \frac{3\mu_0 m^2}{4\pi r^4}\sin(2\alpha) \qquad (7)$$

where F_{mr} and F_{mo} represent the radial and tangential force components of the magnetic interaction force, respectively. r is the distance between the particles, and α denotes the angle between the axis of the two dipoles and their magnetization direction.

Experimental Setup: To actuate the microrobots, we utilized an NdFeB permanent magnet and moved it to provide the desired magnetic field (Fig. S11). The magnetic fields were measured by a magnetometer (Changsha Tianheng Measurement and Control Technology Co., Ltd., China). Microscale glass tubes (inner diameter: 1.3 mm) were utilized to mimic vessels. In ultrasound-guided experiments, we used silicone to wrap plastic tubes to simulate human tissue (Fig. S16). Artificial blood (pH: 7.3, surface tension: 0.042 N/m) was purchased from Chuangfeng Automation Technology Co., Ltd., China. All simulations were conducted using COMSOL software to verify the experiments.

Liquid-Gas Reactions for Biosensing: In biosensing experiments, we produced microrobots composed of CO_2 bubbles. Similar to normal microrobot production, we added 1~2 g microparticles in a small vial (3 mL to 5 mL) without processing. Then, we added approximately 2 mL of Sprite soda (The Coca-Cola Company, USA) to the vial and slowly shook it sideways by hand for 30 seconds (frequency: approximately 1 Hz). CO_2 gas was released and formed many tiny bubbles that adsorbed microparticles. When the bubbles maintained their structures, microrobots composed of CO_2 gas bubbles were successfully produced. Because numerous bubbles are generated during shaking slowly, greatly improving the efficiency of contacts between bubbles and hydrophobic microparticles, we can make plenty of microrobots swiftly. It is a practical method to increase productivity (Fig. S17). Alkaline environments were created by adding NaOH solution (pH: 13). The related chemical reaction is provided as follows:

$$2NaOH + CO_2\ (gas) \rightarrow Na_2CO_3 + H_2O\ (liquid). \qquad (8)$$

It provides an excellent example for further biosensing applications (Fig. S18).

Biological Testing: The utilized mice are male nude mice, 8-week old, obtained from the Animal Research Core of the Faculty of Health Science, University of Macau. Avetin was used for anesthesia and euthanasia of the mice (under the assistance of Ms. Moxin Li with mouse tail vein injection). Approval of all ethical and experimental procedures and protocols was granted by the Research Ethics Committee of the University of Macau under Application No. UMAEC-015-2019 and performed in line with the Animal Protection Act enacted by the Legislative Council of Macao Special Administrative Region under Article 71(1) of the Basic Law. For the cell viability test, after autoclaving, the appropriate volume (1 μL, 2 μL, 5 μL, and 10 μL) of magnetic particles was washed with 1 ml of cell culture medium and then mixed with 200,000 HUVEC or SVEC4-10 endothelial cells (gift from Dr. Xiaoling Xu, Faculty of Health Sciences, University of Macau) and seeded into a 6-well plate. The representative pictures of the co-cultured cells were taken 48 hours of the cell culture. Then, the cells were trypsinized and resuspended as single-cell suspension for cellular viability test using Trypan Blue (STEMCELL Technologies, #07050) according to the manufacturer's instruction (Fig. S12).

Section S2. Analysis of the generation of microrobots

Essentially, magnetic air bubble microrobots are a kind of Pickering bubble whose generation is due to the collective effects of collision, attachment, and balance of relative interactions between the magnetic hydrophobic microparticles and the air bubble (1–5). In fact, this procedure has been widely utilized in floatation for mineral engineering or relative fields (6). To capture the hydrophobic particles by the bubbles, the distance between them should be close enough for related attractive surface interactions to affect them.

The whole capture procedure of particles and bubbles can be divided into three processes. First, the particles experience hydrodynamics far from the bubbles (7). Through several fluidic interactions and gravity, part of the particles can reach the surfaces of bubbles. Then, the surface interactions between particles and bubbles should facilitate breaking the liquid film and forming the three-phase contact line (gas, liquid, and solid) so that the particles can be attached to the bubble's surface. Finally, the particle-bubble aggregate must be stable, where the total attachment interactions should be stronger than the total detachment interactions. These three processes are illustrated in Figure 1.

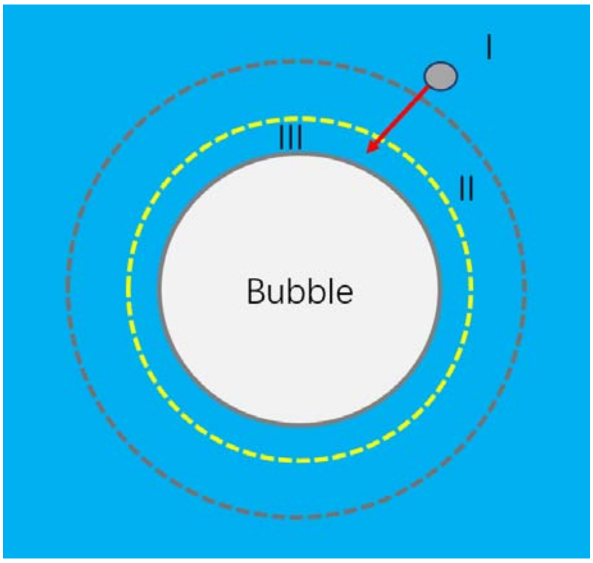

Figure 1. Zone I, Zone II, and Zone III indicate the hydrodynamic zone, diffusiophoretic zone, and surface interaction zone, respectively.

The efficiency of particle capture E_{cap} can be defined as (7):

$$E_{cap} = E_C E_A E_S \quad (1)$$

where E_C, E_A, and E_S denote the collision efficiency, attachment efficiency, and stability of bubble-particle aggregate, respectively. Thus, the sizes of microrobots greatly depend on the sizes of the generated bubbles to adsorb those particles.

Section S3. Analysis of microrobot's physical parameters

The density of the microrobot can be easily defined. The total mass of those microrobots is mainly contributed by those microparticles adsorbed in the air bubble surfaces. To simplify the circulation, the density (ρ_m) of microrobots can be described as:

$$\rho_m = \frac{6\bar{r}_p \rho_p C}{R} \qquad (2)$$

where $\bar{r}_p$, ρ_p, R, and C represent the mean radius of those particles adsorbed on the air bubble surface, the density of the particles, the radius of the microrobot (or the air bubble), and the cover coefficient indicating the encapsulation of particles to bubbles, respectively. Through measurement, statistics, and estimation for the generated microrobots, we can approximate the physical sizes needed for the microrobot to float up naturally. Detailed values are provided in Table 1. Due to the variation in the particle's sizes and shapes, the approximation can only be used as a reference. The analysis can still reveal the relationship between the microrobot's radius and density. In addition, the air bubble can stay stable when the air bubble is partly armored. It means that microrobots are not full of magnetic particles on their surfaces. By applying stronger magnetic fields (100 mT) with changes, we can separate part of the magnetic particles from the air bubble surfaces, which helps to modify further the density of the microrobots (Fig. 2).

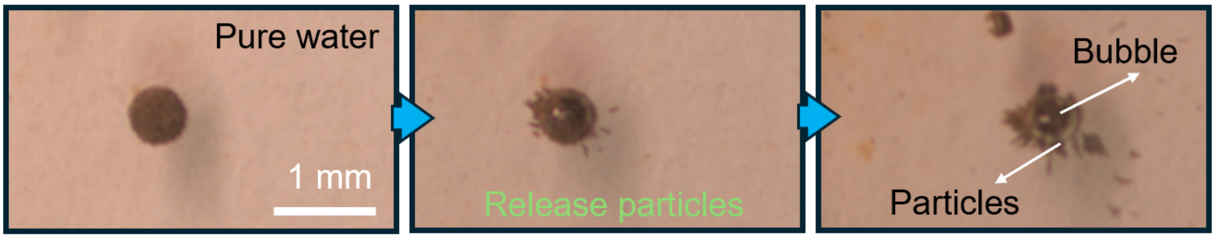

Figure 2. Release part of adsorbed particles to modify the density of microrobots.

Table 1. Detailed values of given parameters for calculation.

Parameter	Value
$\bar{r}_p$	7.5 μm
ρ_p	7.9~8.9 g/cm^3
C	0.9

Section S4. Analysis of the microrobot actuation in fluidic environments

Usually, magnetic microrobots are actuated by magnetic forces and magnetic torques. For magnetic torque actuation, microrobots are rotated to generate propulsion. The magnetic air bubble microrobots can be regarded as microrollers in the rotation motion. In a fluidic environment, microrobot's rotation motion contributes to the hydrodynamics mismatch near the wall, causing translational motion. The hydrodynamic no-slip boundary hinders the microrobot's movement, leading to the mismatch. Detailed fluidic velocity distributions are provided in Fig. S5. However, no translational motion is generated without the necessary contact with the wall. Detailed illustrations and force analysis are provided in Figure 3. We utilized the lubrication theory to describe the forces (F_r) acting on the rotating magnetic microrobot (11).

$$F_r = \pi\mu R^2\omega\left(\frac{4}{5}\ln\frac{R}{d} - 1.516\right) \quad (3)$$

where ω and d indicate the angular velocity and the distance between the microrobot and the wall, respectively. F_{up} is composed of the buoyancy force (F_{rb}), and the electrostatic repulsion force (F_{re}). F_{rg} denotes the gravity. These forces are defined as follows.

$$F_{rb} = \frac{4}{3}\pi R^3\rho_f g \quad (4)$$

$$F_{rg} = \frac{4}{3}\pi R^3\rho_m g \quad (5)$$

$$F_{re} = 4\pi\varepsilon\varepsilon_0 Rk v_1 v_2 e^{-kd} \quad (6)$$

$$F_{up} = F_{re} + F_{rb} \quad (7)$$

where ε and ε_0 represent the permittivity of the medium and the vacuum permittivity, respectively, k^{-1} is the Debye length, v_1 and v_2 denote the zeta potential of the microrobot and the wall, respectively.

F_h is the force caused by the hydrodynamic interactions that can be calculated as viscous drag force:

$$F_d = 6\pi\mu RV\epsilon \quad (8)$$

Then, in the rotation motion, related analysis is provided in detail. The wall is significant in enabling microrobot's movement, which still has some limitations.

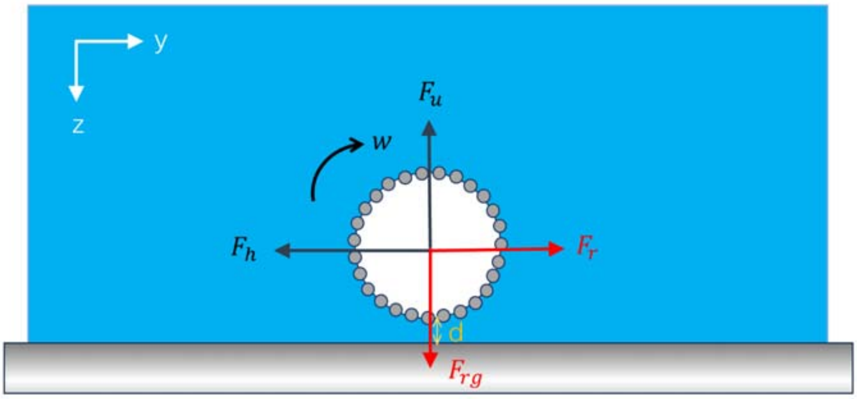

Figure 3. Schematic of force analysis of the microrobot's rotation.

For more straightforward magnetic actuation, we utilized magnetic forces to actuate those microrobots. It performs well in both microrobots near the wall and the water-air surface. Magnetic forces are the only propulsion that can be controlled by moving magnets. The schematic of force analysis is detailed in Figure 4. The magnetic force can be theoretically defined as follows.

$$\mathrm{F}_m = \int_{V_m} (M \cdot \nabla) B \, dV_m \tag{9}$$

where V_m, M, and B denote the volume of the magnetized object (essentially the particles adsorbed on the air bubble surface), the magnetization of the object (Fig. S2), and the flux density of the magnetic field, respectively. Near the water-air interface, the electrostatic repulsion force can be neglected. By tuning the magnetic field density, it can achieve flexible, controlled movement.

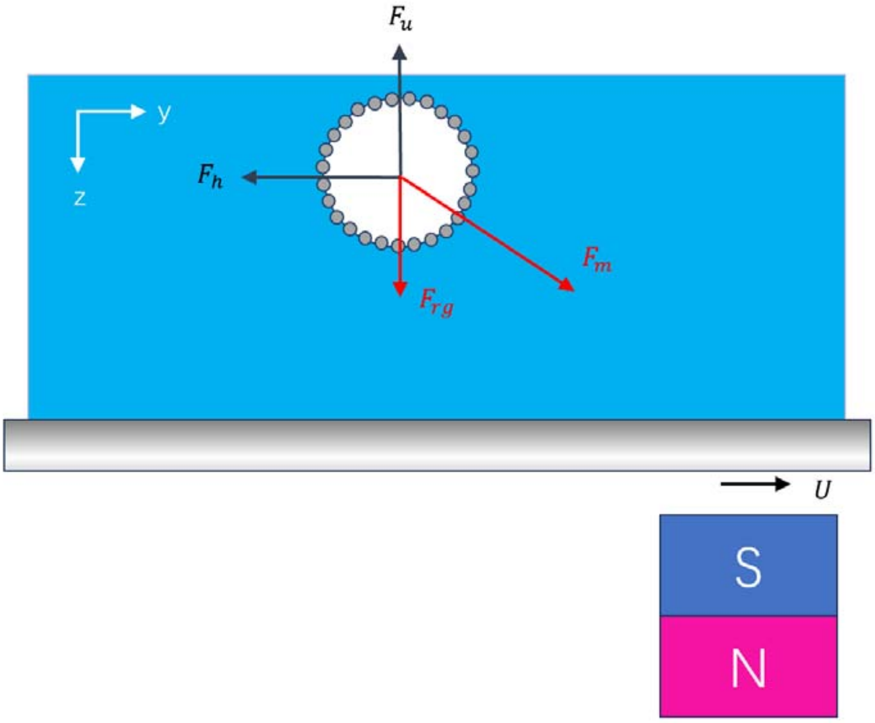

Figure 4. Schematic of force analysis of the microrobot's magnetic force actuation.

Section S5. Analysis of microrobot's stability under magnetic actuation

As a kind of Pickering bubble, our microrobots are much more stable than those normal bubbles. Once microparticles are absorbed on the bubble's surface, high energies are required to remove particles and separate them. The fundamental equation to describe it can be defined as (8):

$$-\Delta E = \pi r^2 \gamma (1 - |\cos\theta|)^2 \qquad (10)$$

where θ, r, and γ denote the contact angle in the aqueous phase, the radius of the particle (i.e., assumed spherical), and the air-water (A/W) interfacial tension, respectively. This indicates that microrobots can stay stable under low-energy conditions.

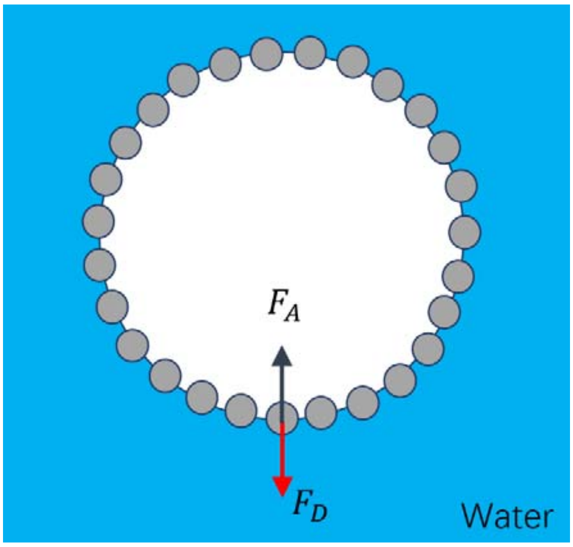

Figure 5. Schematic of force analysis of particles. F_A and F_D indicate the total force for attachment and the total force for detachment, respectively.

A related force condition analysis is conducted to reveal further details of microrobots' stability. For simplification, we only focus on the bubbles and single particles. The attachment forces (F_A) contain buoyancy (F_b), capillary (F_c), and hydrostatic pressure (F_h) induced forces. The detachment forces (F_D) contain gravity force (F_g) and capillary pressure-induced force (F_{cp}) and viscous drag force (F_d). When F_A and F_D reach the balance state, the microrobot will stay stable. All related forces are defined as follows (9):

$$F_A = F_b + F_c + F_h \qquad (11)$$

$$F_D = F_g + F_{cp} + F_d \qquad (12)$$

$$F_b = \frac{4}{3}\pi R_p^3 \rho_f g \qquad (13)$$

$$F_c = 2\pi r_0 \sigma \sin\alpha \qquad (14)$$

$$F_h = \pi r_0^2 \rho_f g h \qquad (15)$$

$$F_g = \frac{4}{3}\pi R_p^3 \rho_p g \qquad (16)$$

$$F_{cp} = P\pi r_0^2 \qquad (17)$$

$$F_d = 6\pi\mu R_p V\epsilon \quad (18)$$

where R_p, ρ_f, ρ_p, and g denote the particle's radius, the density of the fluid, the density of the particle, and the gravitational acceleration, respectively, r_0 is the projected radius of the particle on the three-phase contract plane, σ is the fluid surface tension, α denotes the angle of the bubble surface deviating from the horizontal plane, h represents the height of the surface curvature of the bubble because of the attachment of the particle, P is the internal pressure of the bubble, μ is dynamic fluid viscosity, V denotes the relative velocity of the particle, and ϵ denotes the drag correction factor. Thus, all related forces have been clearly described (Figure 5).

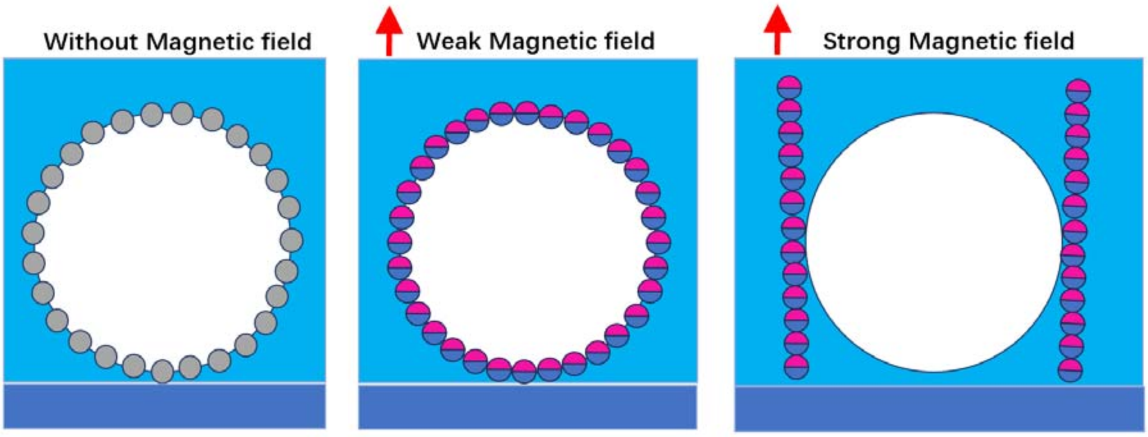

Figure 6. Schematics of the microrobot structure change under different external actuation magnetic fields.

Under magnetic actuation, the introduction of magnetic forces will affect the balance between F_A and F_D, as illustrated in Figure 5. By applying an external magnetic field, those particles are magnetized, which can be regarded as tiny magnets. When the magnetic forces between magnetic particles are sufficient to break the balance, the structures of microrobots will be changed (Figure 6). Then, the interaction force between two particles in a magnetic field can be defined below (10).

$$\boldsymbol{F_{mr}} = \frac{\boldsymbol{3\mu_0 m^2}}{\boldsymbol{4\pi r^4}}(\boldsymbol{3\cos^2\alpha - 1}) \quad (\boldsymbol{19})$$

$$\boldsymbol{F_{mo}} = \frac{\boldsymbol{3\mu_0 m^2}}{\boldsymbol{4\pi r^4}}\boldsymbol{\sin(2\alpha)} \quad (\boldsymbol{20})$$

where F_{mr} and F_{mo} represent the radial and tangential force components of the magnetic interaction force, respectively. r is the distance between the particles, and α denotes the angle between the axis of the two dipoles and their magnetization direction. By analyzing the force conditions, it is obvious to reveal the relationship among those interactions for better utilizing the microrobots. Weak magnetic fields can actuate all the particles so the microrobot can achieve controlled movement. Strong magnetic fields contribute to powerful interaction forces between the particles, where all particles will form a chain-like structure with the direction indicating the external magnetic field. Thus, the inside air bubble is exposed to external fluid environments.

Section S6. Interface interactions between the magnetic air bubble microrobots and various objects

The water-air interface interactions are significant in a microrobot manipulation task. When the structures of microrobots are stable and complete, those particles adsorbed on the surface prevent contact between the air bubble and the external objects. Under this circumstance, the microrobot can be regarded as a micro-roller or micro-ball that can push objects, as detailed in Figure 7A. Applying strong magnetic fields (>100 mT), the air bubble is exposed to external environments, which can capture the hydrophobic objects well, as shown in Figure 7B.

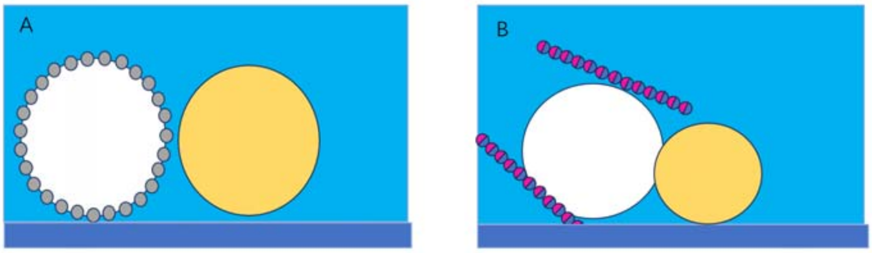

Figure 7. Schematic of the microrobot's contact manipulation.

References

1. P. Amani, R. Miller, A. Javadi, M. Firouzi, Pickering foams and parameters influencing their characteristics. *Adv. Colloid Interface Sci.* **301** (2022), doi:10.1016/j.cis.2022.102606.
2. E. Blanco, S. Lam, S. K. Smoukov, K. P. Velikov, S. A. Khan, O. D. Velev, Stability and viscoelasticity of magneto-pickering foams. *Langmuir*. **29**, 10019–10027 (2013).
3. P. Valadbaigi, R. Ettelaie, A. N. Kulak, B. S. Murray, Generation of ultra-stable Pickering microbubbles via poly alkylcyanoacrylates. *J. Colloid Interface Sci.* **536**, 618–627 (2019).
4. C. Y. Xie, S. X. Meng, L. H. Xue, R. X. Bai, X. Yang, Y. Wang, Z. P. Qiu, B. P. Binks, T. Guo, T. Meng, Light and Magnetic Dual-Responsive Pickering Emulsion Micro-Reactors. *Langmuir*. **33**, 14139–14148 (2017).
5. D. Wu, V. Mihali, A. Honciuc, PH-Responsive Pickering Foams Generated by Surfactant-Free Soft Hydrogel Particles. *Langmuir*. **35**, 212–221 (2019).
6. S. Ding, Q. Yin, Q. He, X. Feng, C. Yang, X. Gui, Y. Xing, Role of hydrophobic fine particles in coarse particle flotation: An analysis of bubble-particle attachment and detachment. *Colloids Surf. A. Physicochem. Eng. Asp.* **662** (2023), doi:10.1016/j.colsurfa.2023.130980.
7. J. Ralston, S. S. Dukhin, The interaction between particles and bubbles, *Colloids Surf. A Physicochem Eng. Asp.*, **151**, 3-14 (1999).
8. F. Martínez-Pedrero, Static and dynamic behavior of magnetic particles at fluid interfaces. *Adv. Colloid Interface Sci.* **284**, 102233 (2020).
9. A. Eskanlou, M. H. Chegeni, M. R. Khalesi, M. Abdollahy, Q. Huang, Modeling the bubble loading based on force balance on the particles attached to the bubble. *Colloids Surf A Physicochem Eng. Asp.* **582** (2019), doi:10.1016/j.colsurfa.2019.123892.
10. Y. Alapan, U. Bozuyuk, P. Erkoc, A. C. Karacakol, M. Sitti, Multifunctional surface microrollers for targeted cargo delivery in physiological blood flow. *Sci. Robot.* **5**, 1–11 (2020).
11. I. Petousis, E. Homburg, R. Derks, A. Dietzel, Transient behaviour of magnetic micro-bead chains rotating in a fluid by external fields. *Lab Chip*. **7**, 1746–1751 (2007).

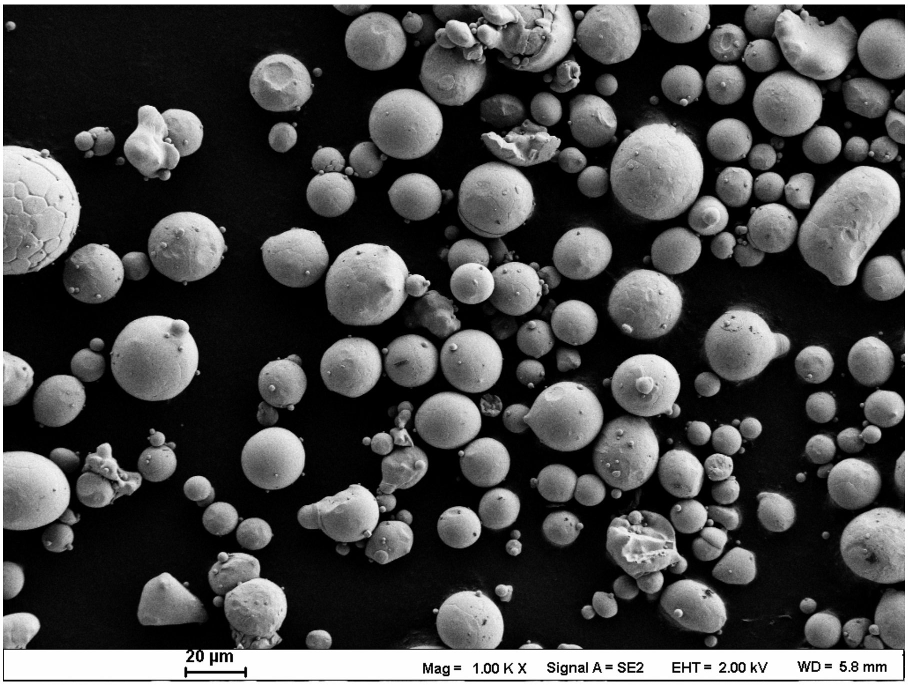

Fig. S1. SEM image of the adopted magnetic microparticles.

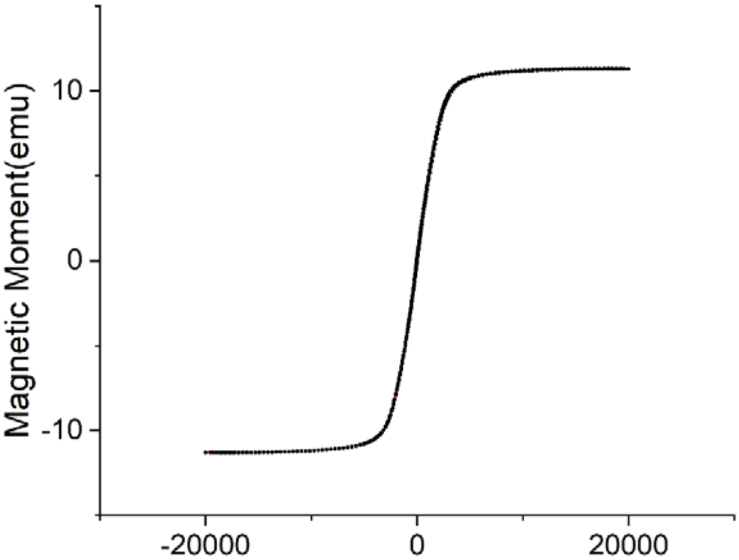

Fig. S2. Magnetic hysteresis loop indicating the microparticle's magnetization properties.

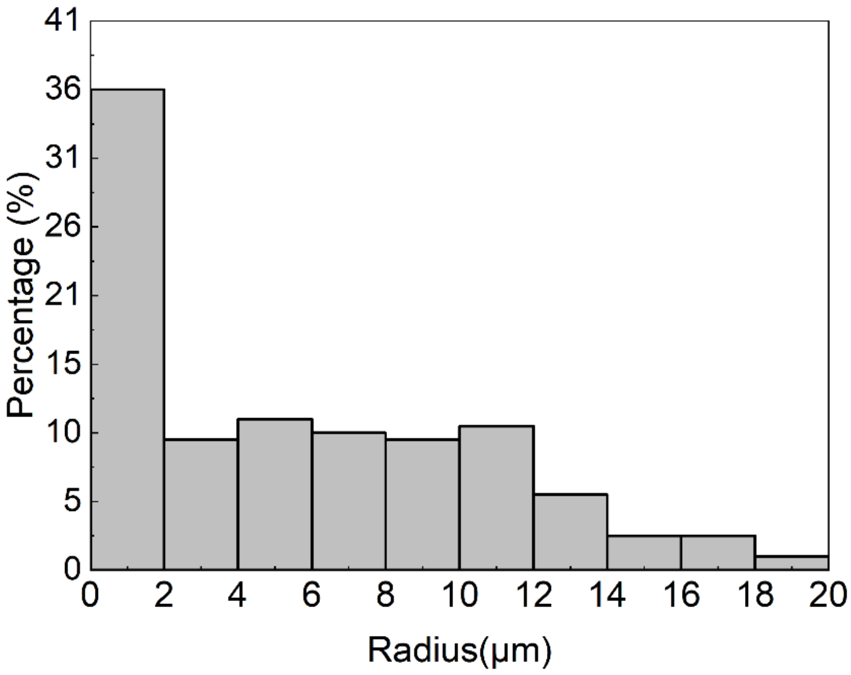

Fig. S3. Radius distribution of the adopted microparticles in Fig. S1.

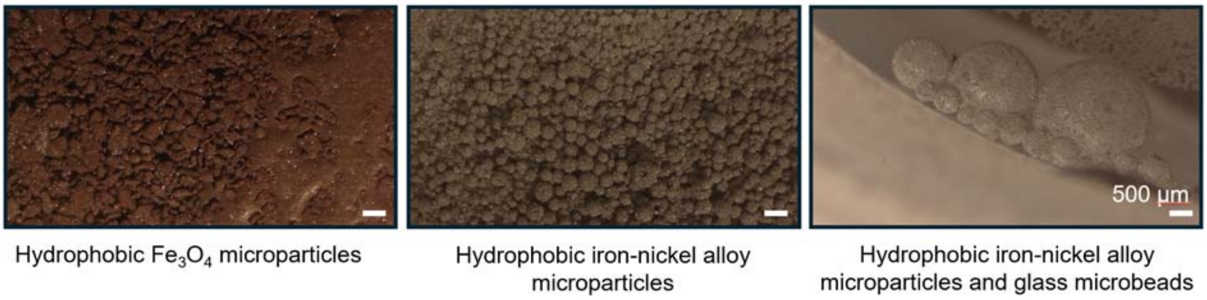

Fig. S4. Utilizing microparticles with different sizes to generate air bubble microrobots.

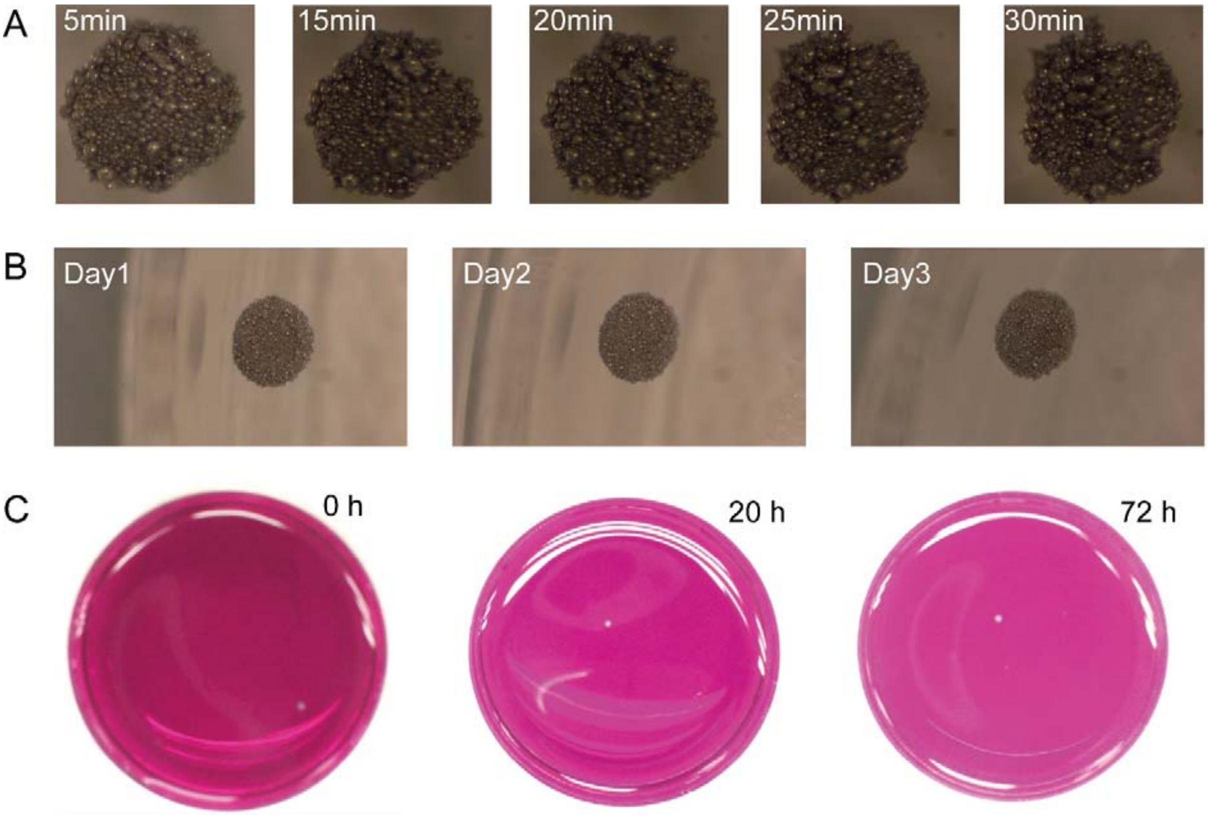

Fig. S5. Microrobots stay stable in various environments. (A) The microrobot can remain stable in acid environments (pH 1.5) for at least 30 minutes. **(B)** The microrobot can stay stable in alkaline environments (pH 12) for over 3 days. **(C)** The microrobot can remain stable in artificial blood for over 3 days.

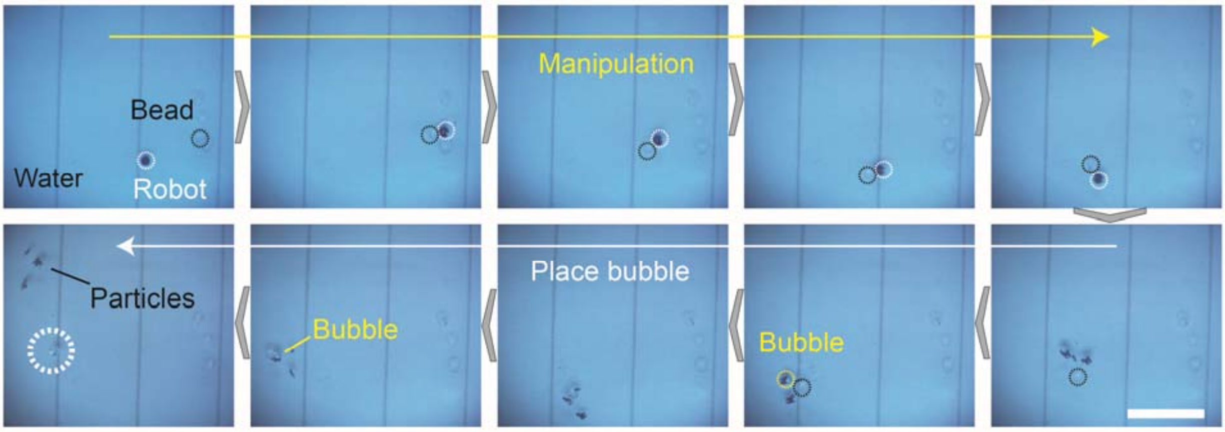

Fig. S6. Microrobot manipulates a glass bead (diameter: 0.8 mm) and places a microbubble on the bead surface. Scale bar: 10 mm.

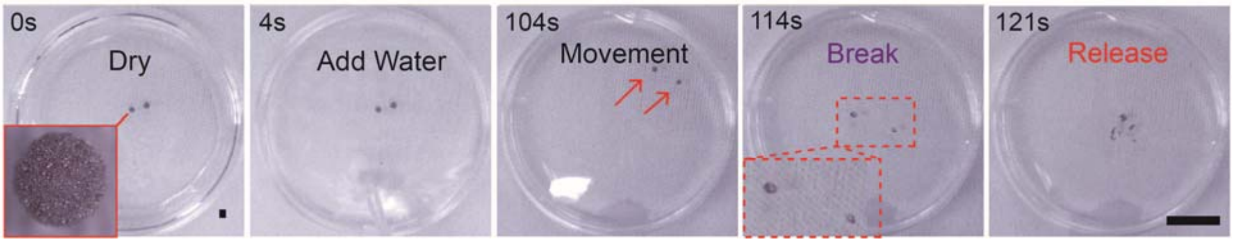

Fig. S7. Microrobots (diameter: around 1 mm) maintain their physical structures in a dry environment and perform with the same properties as in a fluidic environment after adding water. It's a natural air-drying process, around 12 hours. Scale bar: 10 mm. When the hydrophobic particles are fully covered the air bubble, those particles can be regarded as tiles which are tightly constructed. In this condition, those particles can support each other to maintain the structure. However, when those particles are not fully covered the bubble. Those particles are loosely constructed. The existence of bubbles can support the structure. After removing the water and the bubble, the structure cannot be maintained.

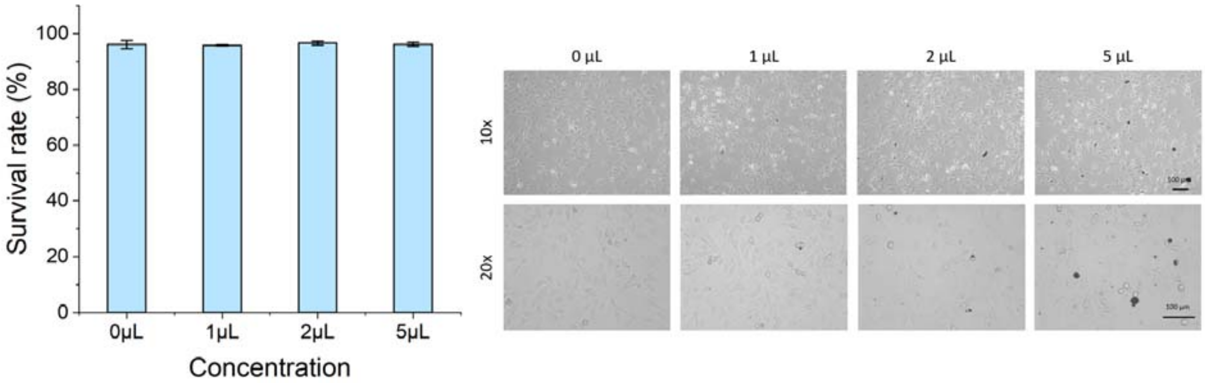

(A) (B)

Fig. S8. Experimental results of biosafety testing. (A) The survival rate of SVEC4-10 cells that were co-cultured with different concentrations of magnetic microparticles for 48 hours. **(B)** Experimental images of utilized cells and magnetic particles.

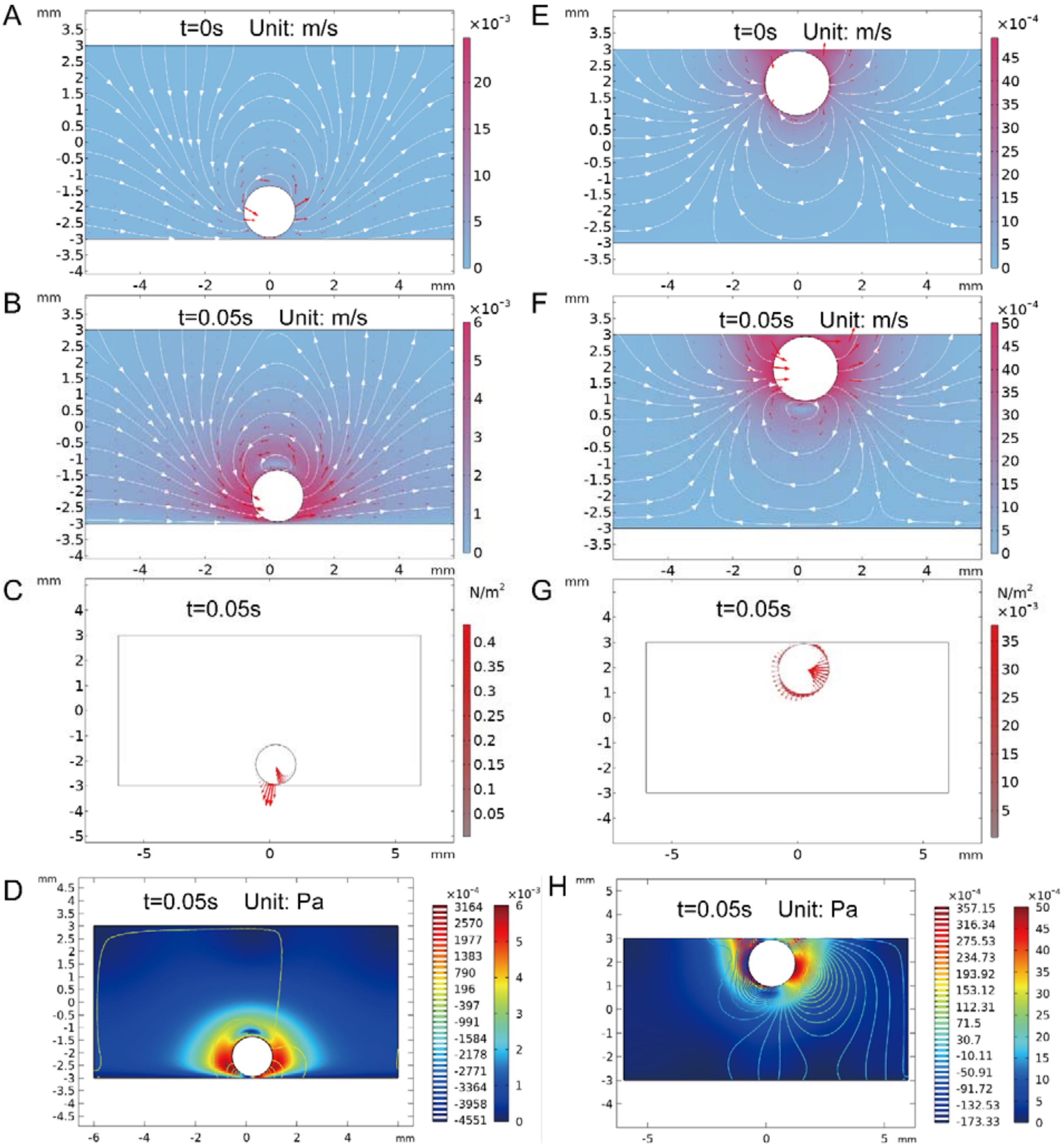

Fig. S9. COMSOL simulation results of the microrobot's parallel movement under different fluidic surroundings. (A)(B)(E)(F) The induced flow distribution is caused by moving microrobots with the same velocity of 5 mm/s at different time in a static fluidic environment. **(C)** and **(G)** indicate the boundary load of different microrobots. **(D)** and **(H)** describe the pressure distributions.

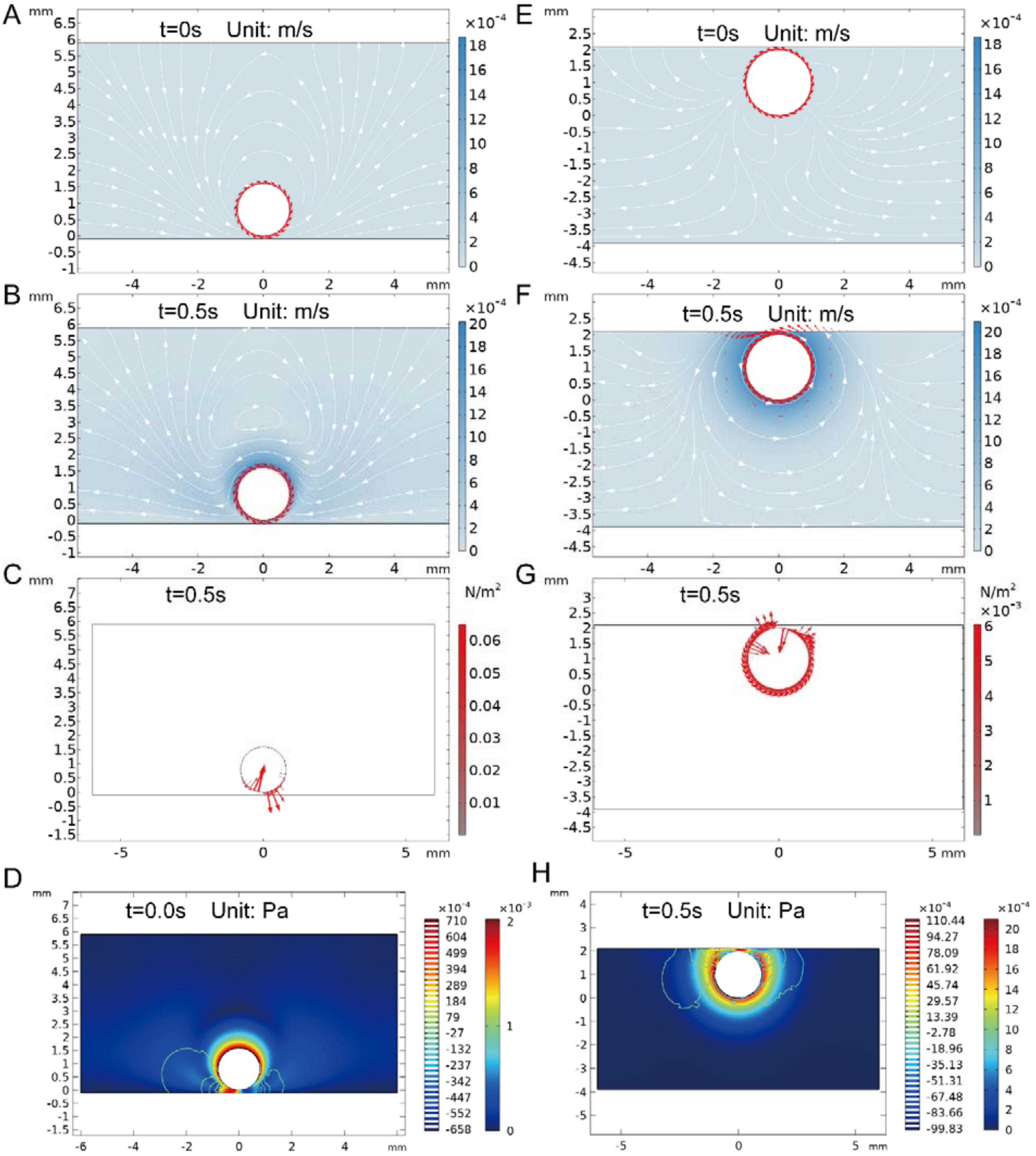

Fig. S10. COMSOL simulation results of the microrobot's self-rotation under different fluidic surroundings. (A)(B)(E)(F) The induced flow distribution is caused by moving the microrobots with the same rotation frequency at different time in the static fluidic environment. These results prove that the interactions near the wall lead to the hydrodynamic mismatch between the rotating microrobot's top and bottom parts. **(C)** and **(G)** indicate the boundary load of different microrobots. **(D)** and **(H)** describe the pressure distributions.

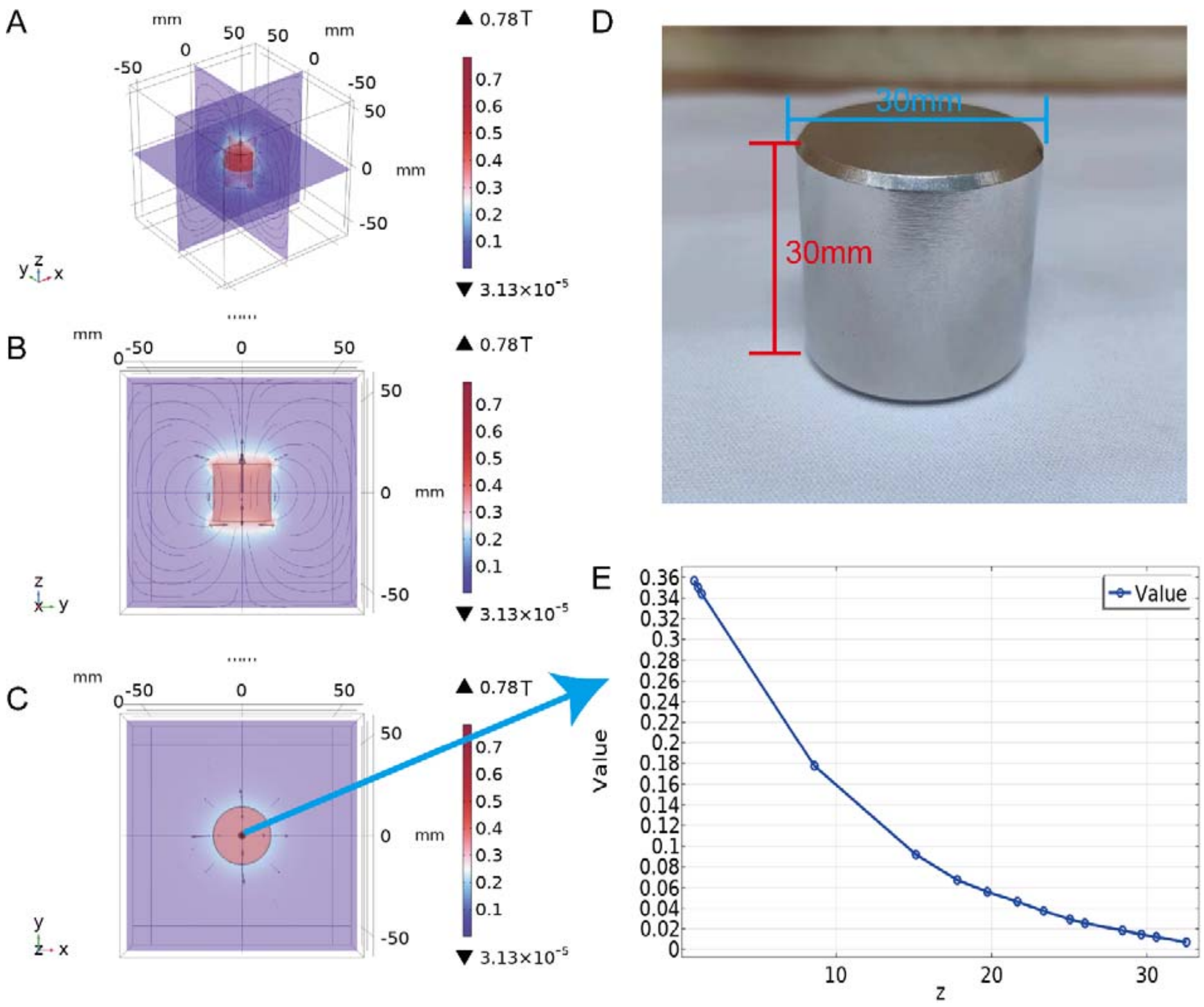

Fig. S11. Detailed information of the utilized NdFeB permanent magnet. (A)-(C) COMSOL simulation results of the magnet. **(D)** Actual image of the utilized magnet. **(E)** Simulated value results indicate the magnetic field density distribution in the z-axis direction at the point in **(C)**. The unit of the value is T, and z denotes the distance from the magnet surface (unit: mm).

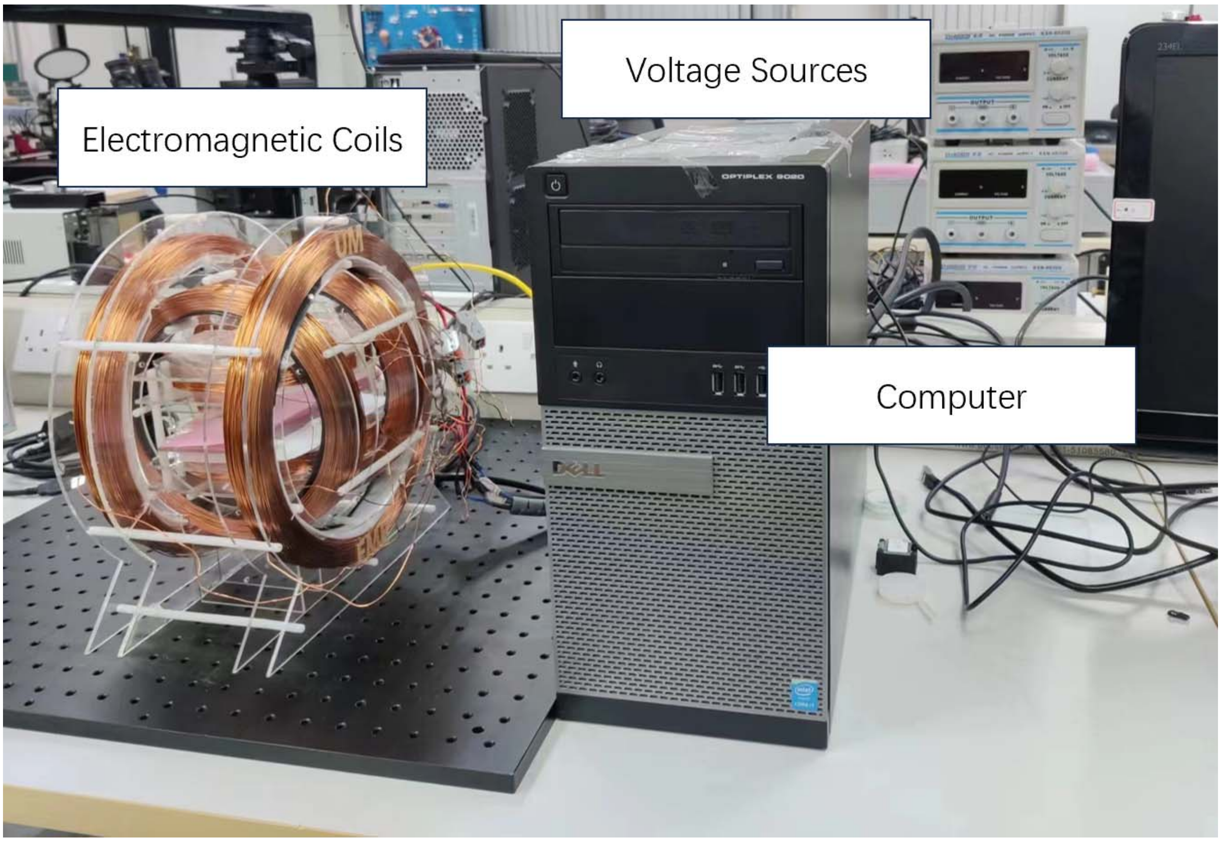

Fig. S12. Electromagnetic coils and control computer to achieve precise movement control for microrobots.

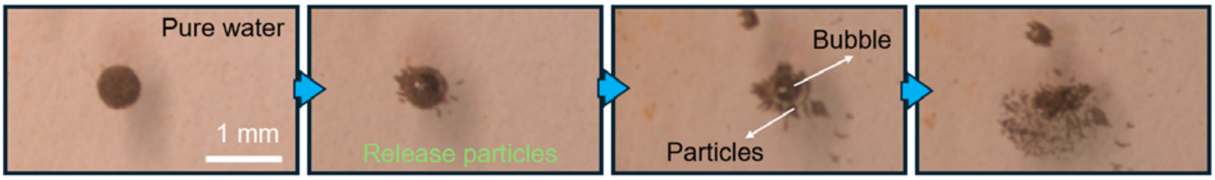

Fig. S13. Wireless magnetically controlled release of magnetic particles.

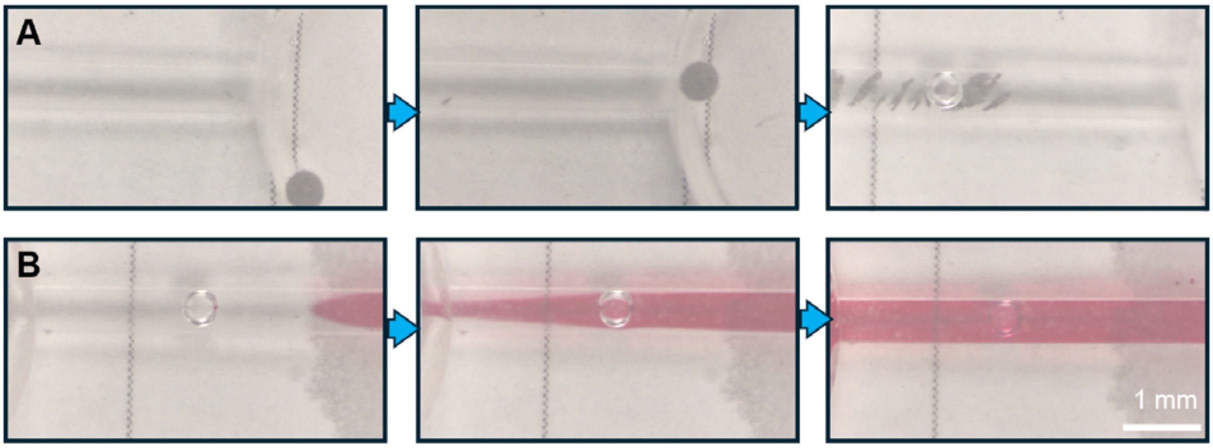

Fig. S14. Controlled bubble release to form potential embolism. (A) Release the bubbe in the channel. (B) The bubble adheres to the channel surface to resist water flow.

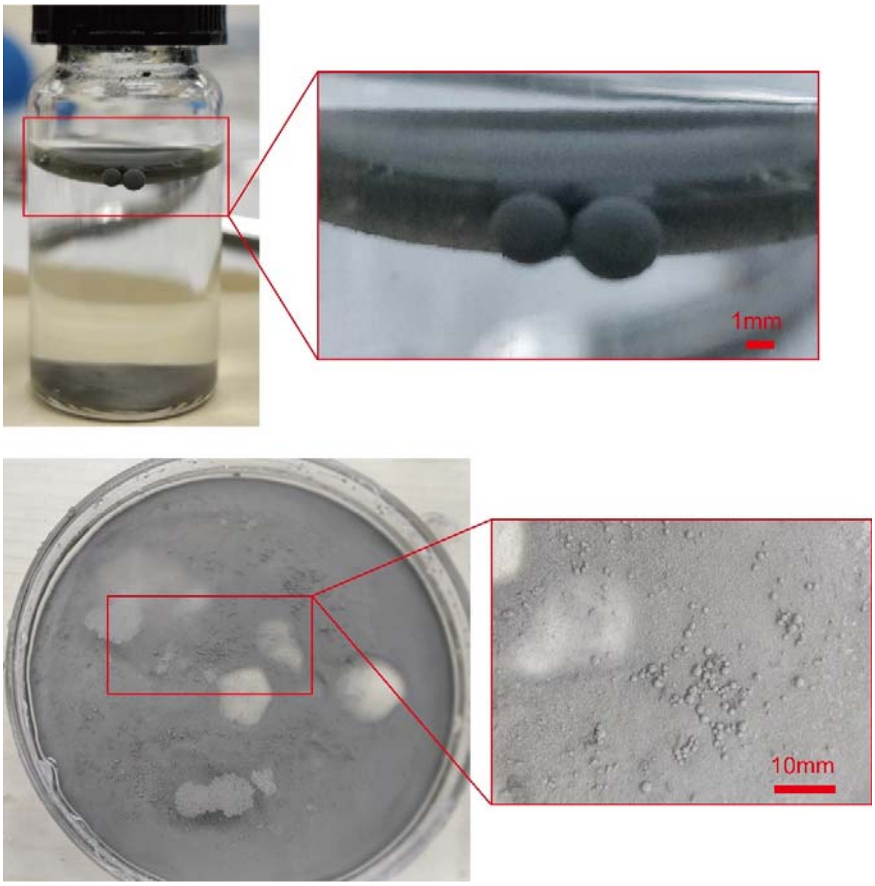

Fig. S15. Images of the generated magnetic air bubble microrobots.

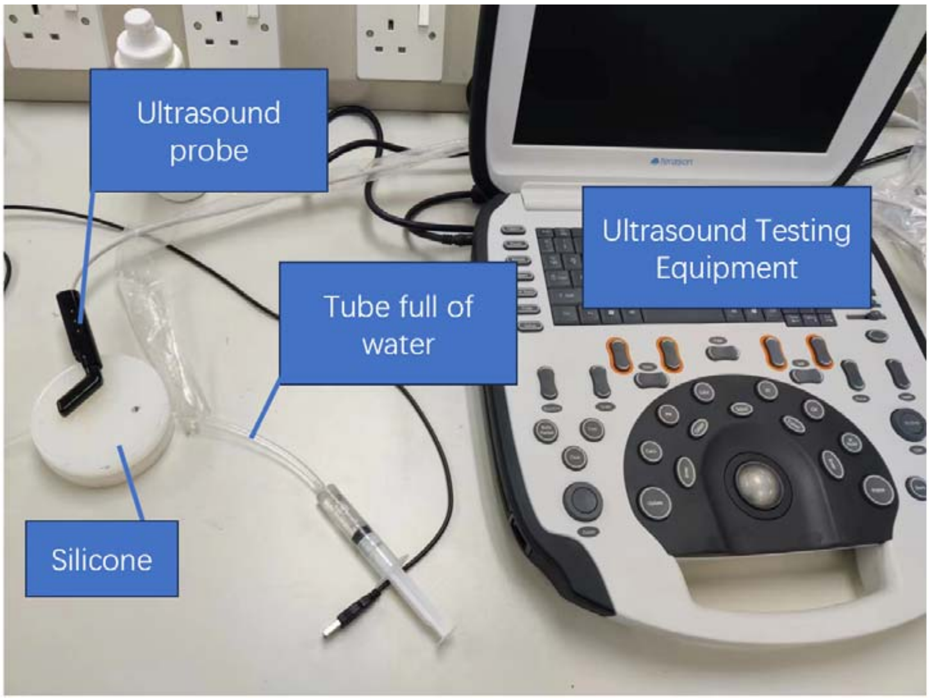

Fig. S16. Experimental setup for ultrasound-based guidance of the microrobot.

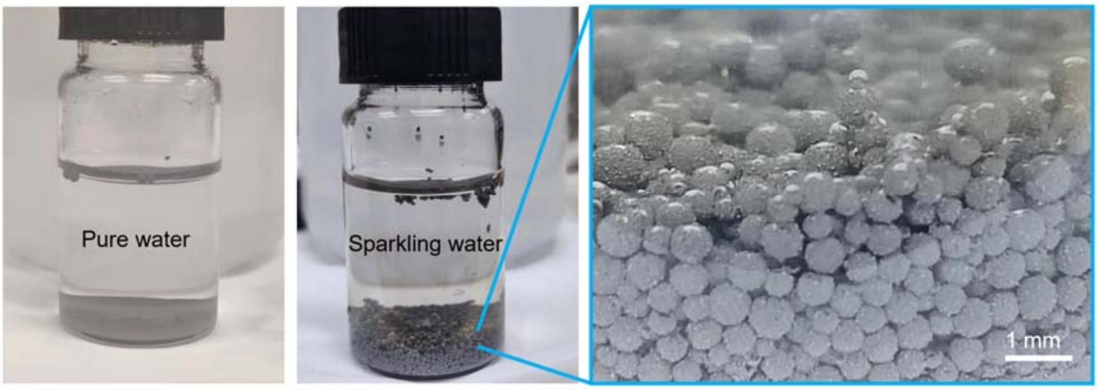

Fig. S17. Due to swift bubble generation by using sparkling water, microrobots are produced more efficiently than utilizing pure water.

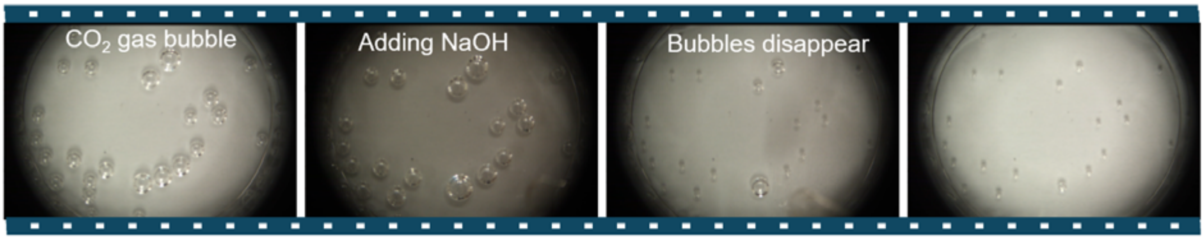

Fig. S18. CO2 bubbles' sizes are sensitive to alkaline environments.
